# Noise Helps Optimization Escape From Saddle Points in the Synaptic Plasticity

**DOI:** 10.3389/fnins.2020.00343

**Published:** 2020-04-29

**Authors:** Ying Fang, Zhaofei Yu, Feng Chen

**Affiliations:** ^1^Department of Automation, Center for Brain-Inspired Computing Research, Tsinghua University, Beijing, China; ^2^Beijing Innovation Center for Future Chip, Beijing, China; ^3^Beijing Key Laboratory of Security in Big Data Processing and Application, Beijing, China; ^4^National Engineering Laboratory for Video Technology, School of Electronics Engineering and Computer Science, Peking University, Beijing, China

**Keywords:** noise, strict saddle, synaptic sampling, synaptic plasticity, free energy

## Abstract

Numerous experimental studies suggest that noise is inherent in the human brain. However, the functional importance of noise remains unknown. n particular, from a computational perspective, such stochasticity is potentially harmful to brain function. In machine learning, a large number of saddle points are surrounded by high error plateaus and give the illusion of the existence of local minimum. As a result, being trapped in the saddle points can dramatically impair learning and adding noise will attack such saddle point problems in high-dimensional optimization, especially under the strict saddle condition. Motivated by these arguments, we propose one biologically plausible noise structure and demonstrate that noise can efficiently improve the optimization performance of spiking neural networks based on stochastic gradient descent. The strict saddle condition for synaptic plasticity is deduced, and under such conditions, noise can help optimization escape from saddle points on high dimensional domains. The theoretical results explain the stochasticity of synapses and guide us on how to make use of noise. In addition, we provide biological interpretations of proposed noise structures from two points: one based on the free energy principle in neuroscience and another based on observations of *in vivo* experiments. Our simulation results manifest that in the learning and test phase, the accuracy of synaptic sampling with noise is almost 20% higher than that without noise for synthesis dataset, and the gain in accuracy with/without noise is at least 10% for the MNIST and CIFAR-10 dataset. Our study provides a new learning framework for the brain and sheds new light on deep noisy spiking neural networks.

## 1. Introduction

It has been observed that noise permeates everywhere in the nervous system and affects all aspects of brain function (Mori and Kai, 2002; Fellous et al., 2004; Faisal et al., 2008). On the other hand, it has been proposed that action, perception, and learning in the brain such as attention, memory, neural coding, and evolution, can be understood as an optimization process (Friston, 2010). Both noise and optimization are prevalent in the nervous system. So, is there a close relationship between the two? What precisely is the nature of noise that helps the brain compute optimally? In this paper, we argue that typically the answer is YES.

In recent years, many studies have provided insight into which noise structures are present, and how noise affects the structure and function of the nervous system. As far as we know, there are mainly three noise models in stochastic neural circuit computations. The first model is based on the leaky integrate-and-fire (LIF) model. Although LIF models have been extensively applied in biological spiking neural networks, they are still deterministic. Some researchers add a Brownian noise on the potential of IF neurons for better agreement with experimental observations (Soula et al., 2004; Burkitt, 2006; Cessac, 2010, 2011). Brownian noise helps them characterize generic behaviors by exploring a large number of parameters. However, these researchers did not further study other benefits of noise. The second model is based on the mean-filed theory. The mean filed of one neuron represents its effect on the whole neural network. They add noise in the neuron's behavior by assuming a neuron has an instantaneous firing probability in any time step (Galves and Löcherbach, 2013, 2016; Larremore et al., 2014; Duarte et al., 2015). This firing probability *P*_*F*_(*V*) is a function of membrane potential *V*. The mean field depends on the synaptic weights and firing probabilities *P*_*F*_(*V*) from interconnected neurons. Therefore, they simplify the analysis and simulation of noisy spiking neural networks in the mean-field calculation. However, this model groups all sources of noise into a single firing function and is therefore agnostic about the origin of noise. As a result, it is difficult to decompose explicit noise terms from the model, which is a bad thing for the mathematical analysis of noise. The third model is based on sampling. General results from statistical learning theory suggest that both brain computations and brain plasticity should be understood as probabilistic inference (Knill and Pouget, 2004; Pouget et al., 2013). These results have provided insight into how noise plays an essential role in the networks of spiking neurons. Based on Boltzmann machines, Maass (2014) propose that knowledge can be stored in probabilistic distributions of network states, and noise enables networks of spiking neurons to carry out probabilistic inference through MCMC sampling. The sample of this model is the state of neurons and the noise results from the ion channels of excitable membranes. Based on Langevin sampling, Kappel et al. (2015, 2018) analyzed continuously ongoing synapse dynamics and noise endows networks to compensate for internal and external changes automatically in the local plasticity mechanisms. The sample of this model is the state of synapses, and the noise results from the synaptic transmission. In the work by Kappel et al. (2018) in particular, they discuss the impact of different temperatures on learning performance, where the strength of stochasticity can be scaled by the temperature. Results show that good performance was achieved for a range of temperature values and temperatures that were too low (such as without noise) or too high impaired learning. They provide a short explanation through the perspective of an analogy of simulated annealing. However, they did not provide a rigorous theoretical analysis for the noise mechanism. In conclusion, although researchers using the sampling model have claimed that the benefit of noise is a functional part of sampling, to perform probabilistic inference, they do not provide a detailed mathematical analysis of noise and do not study which noise structure is involved, or how it enhances the computation power of spiking neural networks.

In summary, in theoretical neuroscience research, the extent to which noise is biologically present and how noise improves computation performance in the brain has rarely been addressed. Based on the synaptic sampling model (Kappel et al., 2015), we give a detailed mathematical analysis of noise in spiking neural networks and try to explain why our brain benefits from noise. Here, we can generally assume that noise type is fluctuations in synaptic transmission because the proposed noise has an important role in synaptic plasticity. There are many sources of noise in synaptic transmission, such as stochastic molecular diffusion (Holcman et al., 2005), short-term plasticity (Abbott and Regehr, 2004), and synaptic neurotransmitter release (Branco and Staras, 2009). Therefore, we make no assumptions about the concrete sources of noise. Next, we will sketch the noise mechanism and try to bridge the gap between neuromorphic computing and machine learning.

According to the free-energy principle in neuroscience, we propose a biologically plausible noise structure and prove that such noise helps optimization escape from bad saddle points in the brain computation and brain plasticity. First, we propose that one of the essential roles of noise is to improve optimization and prove that the noise mechanisms of improving optimization satisfy the strict-saddle condition of spiking neural networks. The main bottleneck in optimization is that gradient updates are trapped in exponentially more saddle points instead of local minima (Fyodorov and Williams, 2007; Dauphin et al., 2014). Under the so-called strict saddle property, gradient descent with noise will escape from bad saddle points and lead to efficient optimization (Ge et al., 2015). The importance of adding perturbations for efficient non-convex optimization has been justified in many machine applications, including deep learning (Du et al., 2017; Jin et al., 2017). We prove that such noises make spiking neural networks satisfy strict saddle properties by changing the curvature of the landscape in the network parameter space, especially in the area near the saddle points. In other words, noise helps spiking neural networks build appropriate Hessian constructions, and optimization can utilize enriched curvature information of the node in the direction without ever calculating or storing the Hessian itself. Second, the proposed noise in the brain theoretically minimizes the free energy of noise signals. In neuroscience, any self-organizing system at equilibrium must minimize its free-energy to resist disorder (Friston, 2010; Joffily and Coricelli, 2013; Apps and Tsakiris, 2014; Colombo and Wright, 2018). Since free energy can be expressed by long-term average self-information of sensory signals, such as mean square error, we prove that such a particular form of noise comes from minimum mean square error estimation. Third, such noise satisfies the fundamental biological characteristic. It is popular to use Brownian motion to describe continuous random fluctuations in spiking activities (Tuckwell, 1988; Cateau and Fukai, 2003; Câteau and Reyes, 2006; Nobuaki et al., 2009). Compared with traditional Brownian motion, the difference is that the standard deviation of our noise has a positive correlation with dendritic spine size. Moreover, it has been observed that larger spines show the most diverse changes in CA1 pyramidal neurons (Nobuaki et al., 2009). Our noise has a greater standard deviation for larger spines and is hence biologically plausible. Finally, our noisy spiking neural network can also be extended to multi-layer neural networks and obtains better performance. It is generally believed that deep neural networks can learn more complex representations and has shown remarkable success in diverse fields (LeCun et al., 2015). As an example, we realize three-layer noisy spiking neural networks based on gradient back-propagation, which provides a possibility for the realization of large-scale deep networks.

This paper is organized as follows. In section 2, we introduce some complex concepts, such as synaptic sampling and the saddle point problem in non-convex optimization. In section 3, we demonstrate how noise helps optimization escape from saddle points in the neural dynamics and give a proof of sketch on the “strict saddle” condition for synaptic sampling. In section 4, we will explain why the proposed noise structure is biologically plausible from two points: origin from the free-energy principle in neuroscience and consistency with biological observation. In section 5, we derive the learning rule for multi-layer spiking neural networks based on gradient back-propagation for better learning abilities. In section 6, numerical simulations are presented and analyzed. In section 7, we highlight the main contributions of this work and discuss some related open problems. Detailed theorem derivations are deferred to in Appendices 1, 2, 3 (Supplementary Presentation 1).

## 2. Preliminaries

### 2.1. Spiking Neural Networks and Hebb Rule

Spiking neural networks (SNNs) is one of the brain-inspired computing models. Its spike-based coding tends to represent more complex information due to spatio-temporal dynamics. In addition, its computation occurs only when the unit in networks receives a spike signal. Such event-driven property is consistent with emerging neuromorphic hardware. Therefore, SNNs have great potential for energy-efficient processing on neuromorphic hardware (Deneve, 2008; Merolla et al., 2014).

In experimental neuroscience, changes of synaptic strength are called synaptic plasticity. The Hebb rule describes how the strength between pre- and postsynaptic neurons should be modified in synaptic plasticity. It is informally summarized as “Cells that fire together, wire together.” Spike-timing-dependent plasticity (STDP) is one of the Hebbian learning methods. The strength and direction of learning depends on the timing difference between pre- and postsynaptic spikes (Bi and Poo, 1998; Gerstner and Kistler, 2002; Sjöström et al., 2002).

### 2.2. Synaptic Sampling

Network plasticity by maximum likelihood has been studied in many ways. The inputs ***x*** impinge on the network from its environment. By maximizing the likelihood of the inputs, the network parameters **θ** are adjusted to encode the input information. That is to say, maximizing the likelihood, is to fit the resulting internal model to the inputs as best as possible. However, the model tends to produce overfitting, thereby reducing generalization capabilities. Furthermore, without any prior distribution, it responds slowly to perturbations. The solution to such a challenge is how the posterior distribution of weights can be represented and learned in neural dynamics. Based on stochastic differential equations, Kappel et al. (2015) solve this challenge by sampling from posterior distribution *p*_*N*_(**θ**|***x***). This model defined by Equation (1) is referred to as synaptic sampling. Furthermore, they only understand noise as a functional aspect of learning because it helps the network sample from posterior distributions. However, when this model is used for classification with a standard Gaussian noise, it is difficult to find a reasonable minimum due to the saddle point problem, which will be introduced next.

(1) $$\begin{array}{l} d\theta_{ki}=b\left(\frac{\partial \log p_{S}\left(\theta \right)}{\partial \theta_{ki}}+\frac{\partial \log p_{N}\left(x|\theta \right)}{\partial \theta_{ki}}\right)dt+bdW_{ki} \end{array}$$

### 2.3. Saddle Point Problem

Critical points (i.e., minima, maxima, saddle points) are often surrounded by error plateaus of small curvatures, and hence are attractive for the gradient-based learning process. However, as gradient-based algorithms only depend on gradient information, they often mistake saddle points for local minima or maxima. Moreover, it is generally believed that a high-dimensional error functions are likely to have saddle points rather than local minima because the number of saddle points dominate over local minimum exponentially with increasing dimensions (Fyodorov and Williams, 2007; Dauphin et al., 2014). Therefore, gradient-based algorithms are particularly sensitive to saddle point problems.

**Figure 1:**
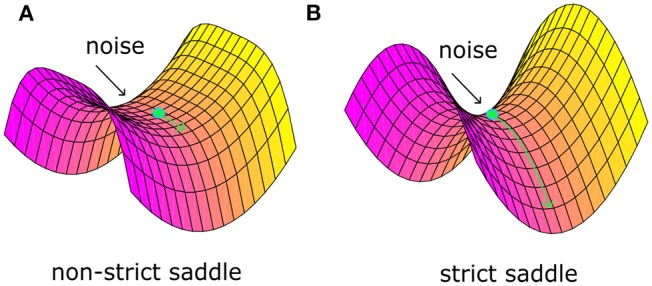
The non-strict saddle point **(A)** and the strict saddle point **(B)**.

Recently, Ge et al. (2015) identified a “strict saddle” condition, which guarantees that stochastic gradient descent can escape from the saddle points quickly (see Theorem 6 in work Ge et al., 2015). Note that a twice differentiable function *f*(θ) is a strict saddle, if all its local maxima have ∇^2^*f*(θ) < 0 and all its other stationary points satisfy $\lambda_{max}\left(\nabla^{2}f\left(\theta \right)\right)>0$. Note that λ_*max*_ defines the maximum eigenvalue. In fact, the “strict saddle” condition guarantees that there will be at least one descent direction in the small neighborhood of saddle points, not a plain area. For example, Figure 1A shows one non-strict saddle point. The area around it is plain and it would be very tough for optimization to escape from such a bottleneck even with noise. Figure 1B shows one strict saddle point. There are at least one descent direction and it will take little time to escape with noise. Based on the above theory, we propose a sufficient condition that noise should satisfy and argue that noise plays a critical role in the brain optimization process.

## 3. “Strict Saddle” Condition for Synaptic Sampling

**Figure 2:**
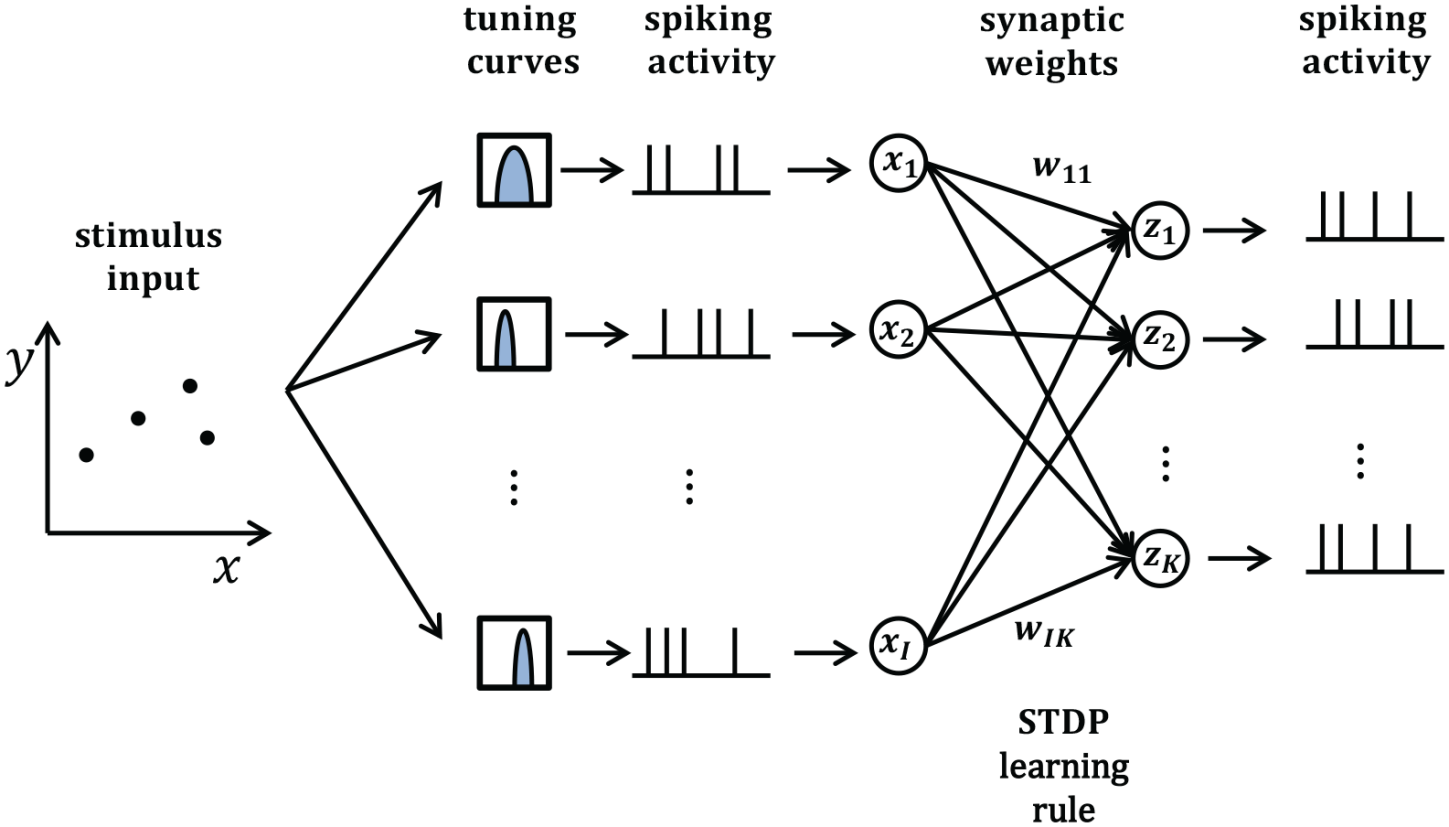
Architecture of the networks whose dynamics are modeled by Equation (3).

In this section, we will take synaptic sampling neural networks as an example and demonstrate how noise improves optimization in neural dynamics. We study the effect of noise on the synaptic sampling defined in Equation (1) for classification. As Figure 2 shows, in the spike-based Winner-Take-All (WTA) circuit, input neurons tune *n*_*th*_ stimulus to 200-ms long spiking activities ***x*^*n*^** according to tuning curves. Given the *n*_*th*_ stimulus, the input *x*_*i*_(*t*) is expressed by the summation of excitatory postsynaptic potentials (EPSPs) on neurons *i* in Equation (2).

(2) $$\begin{array}{l} x_{i}\left(t\right)=\Sigma_{f}\epsilon \left(t-t_{i}^{\left(f\right)}\right) \end{array}$$

where $t_{i}^{\left(f\right)}$ denotes the spike times of input neuron *i* and ϵ is the response kernel for spike input, i.e., the shape of the EPSP (Kappel et al., 2015). The corresponding instantaneous firing rate ρ_*k*_(*t*) of neurons *k* depend exponentially on the membrane potential *u*_*k*_(*t*). In this case, neural networks output a 200-ms spiking pattern ***z*^*n*^** and the neuron which spikes most indicates the possible label. Synaptic sampling is then applied to *K***x***I* synapses. The learning goal in Equation (1) becomes the posterior distribution p^*^(**θ**|*J*)defined by *p*_*S*_(**θ**) * *p*_*N*_(*J*|**θ**). *p*_*N*_(*J*|**θ**) measures the degree of network fitting to the classification. The detailed definition is shown in Table 1. The synaptic sampling rule (Equation 1) yields for this model.

(3) $$\begin{array}{l} d\theta_{\text{ki}}=b(\frac{1}{\sigma^{2}}\left(\mu -\theta_{\text{ki}}\right)+\sum_{n=1}^{N}w_{\text{ki}}\left(x_{i}^{n}-\alpha e^{w_{\text{ki}}}\right)(\Theta \left\{h_{k}^{n}\right\}\\ -S_{k}(t)))dt+bdW_{ki} \end{array}$$

In Equation (3), the component $\left(x_{i}^{n}-\alpha e^{w_{\text{ki}}}\right)\left(\Theta \left\{h_{k}^{n}\right\}-S_{k}\left(t\right)\right)$ of likelihood differential term is a simplified version of STDP (spike timing-dependent plasticity) (Habenschuss et al., 2013; Nessler et al., 2013). Biological studies on STDP show that the timing difference between pre- and post-synaptic spikes decide the strength and direction of learning (Bi and Poo, 1998; Sjöström et al., 2002). When a presynaptic spike comes before a postsynaptic spike, *x*_*i*_ is large and the term $\left(x_{i}^{n}-\alpha e^{w_{\text{ki}}}\right)$ is positive at the time of the postsynaptic spike. Therefore, the term $\left(x_{i}^{n}-\alpha e^{w_{\text{ki}}}\right)$ leads to potentiation. When a presynaptic spike comes after a postsynaptic spike, *w*_ki_ is large and the term $\left(x_{i}^{n}-\alpha e^{w_{\text{ki}}}\right)$ is negative at the time of the postsynaptic spike. Therefore, the term $\left(x_{i}^{n}-\alpha e^{w_{\text{ki}}}\right)$ leads to depression. In addition, the intensity of potentiation is inversely correlated with synaptic weights. It is consistent with experimental STDP studies (Habenschuss et al., 2013; Nessler et al., 2013).

**Table 1:** Definitions of the main mathematical symbols used in this paper.

**x^*n*^**	Vector of the *n*_*th*_ input variables $\left\{x_{1}^{n},\ldots ,x_{I}^{n}\right\}$
**y^*n*^**	Vector of the *n*_*th*_ hidden variables $\left\{y_{1}^{n},\ldots ,y_{J}^{n}\right\}$
**z^*n*^**	Vector of the *n*_*th*_ output variables $\left\{z_{1}^{n},\ldots ,z_{K}^{n}\right\}$
**h^*n*^**	Vector of the label $\left\{h_{1}^{n},\ldots ,h_{K}^{n}\right\}$
**w**	Vector of all synaptic weights $w_{ki}=e^{\left(\theta_{ki}-\theta_{0}\right)}$ from input neuron *i* to network neuron *k*
**θ**	Vector of all synaptic parameters {θ_*ki*_, *k* ≤ *K, i* ≤ *I*}
***p*_*S*_(θ)**	Structural constraints following $N\left(\mu ,\sigma^{2}\right)$
***p*_*N*_(*J*|θ)**	Likelihood function with the form of cross-entropy
	**$\log p_{N}\left(J|\theta \right)=\sum_{n=1}^{N}\sum_{k=1}^{K}\Theta \left\{h_{k}^{n}\right\}\log p\left(z_{k}^{n}|x^{n},\theta \right)$**
**$p_{N}\left(x^{n}|\theta \right)$**	Poissonian distributions of spikes parameterized by $\alpha e^{w_{ki}}$
***dW*_*ki*_**	Stochastic time course of the parameter θ_*ki*_
**$\Theta \left(h_{k}^{n}\right)$**	Heaviside step function
***S*_*k*_(*t*)**	The spike train of the neuron *z*_*k*_

We show that when the noise takes a certain form, synaptic sampling networks for classification satisfy the “strict saddle” condition and leads to efficient optimization. Note that if the noise is just standard normal distribution, which is a popular choice for stochastic differential equations, networks will not satisfy such property.

****Theorem** 1 (sufficient condition)**. *Given the *n*_*th*_ input sample *X*^*n*^, output *S*^*n*^ = *g*(*X*^*n*^, *W*) is a firing rate vector in the synaptic sampling networks and *W* represents adjustable parameters with internal noise dW. In the classification setting, the output *S*^*n*^ can be interpreted as the scores or probabilities of each class, or as the recognized class label of input sample *X*^*n*^. A loss function ϕ(*H*^*n*^, *g*(*X*^*n*^, *W*)) measures the discrepancy between the desired output for input *X*^*n*^, and the output *S*^*n*^ = *g*(*X*^*n*^, *W*) computed by the networks. One objective function *f*(*W*) =* 𝔼*(ϕ(*H, g*(*X, W*))) is average loss function ϕ(*H*^*n*^, *g*(*X*^*n*^, *W*)) over a set of labeled examples {(*X*^1^, *H*^1^), …(*X*^*N*^, *H*^*N*^)}. The supervised learning problem is to find the local minimum *W* of objective function *f*(*W*). if the internal noise dW satisfies Equation (4), function *f* is strict saddle.*

(4) $$\begin{array}{l} dW=N\left(0,N\alpha e^{w}\right)dt \end{array}$$

*Proof sketch of Theorem*. There are mainly two difficulties in the proof of Theorem 1: how to transfer noise distribution to the computable function and how to prove $\lambda_{\max}\left(\nabla^{2}f\left(\theta \right)\right)>0$ according to the definition of strict saddle condition in section 2. For the first difficulty, due to the Gauss property *p*{|*x* − μ| < σ} = 0.6826 and ${\frac{w_{ki}e^{w_{ki}}}{\sqrt{e^{w_{ki}}}}|}_{w_{ki}\to 0}=0$, $\pm N\alpha w_{ki}e^{w_{ki}}$ represents the general characteristic of noise distribution appropriately. Therefore, it is plausible to refer to $\left(\underset{n}{\sum }\alpha w_{\text{ki}}e^{w_{\text{ki}}}\left(S_{k}\left(t\right)-\Theta \left\{h_{k}^{n}\right\}\right)\right)dt$ as the noise distribution $dW_{\text{ki}}$ in the computation. For the second difficulty, according to the definition of strict saddle condition in section 2, the sufficient condition of strict saddle property is $\lambda_{\max}\left(\nabla^{2}f\left(\theta \right)\right)>0$. However, it is difficult to compute λ_max_ directly. In fact, it is convenient to compute a stronger condition, i.e., ∑λ(∇^2^*f*(θ))>0. According to the equation about trace of *n* × *n* matrix A: *tr*(*A*) = λ_1_+…+λ_*n*_, we just concentrate on the diagonal elements of the Hessian matrix. For computational convenience, we convert the derivative of θ to *w* according to the chain rule. We get that ∇*f*(θ) = 0⇔∇*f*(*w*) = 0 and $\underset{k}{\sum }\underset{i}{\sum }\nabla f\left(\theta_{ki}\right)\geq 0\Leftrightarrow \underset{k}{\sum }\underset{i}{\sum }\left(\nabla f\left(w_{ki}\right)\right)w_{ki}^{2}\geq 0$. According to the equation about trace of matrix M: *tr*(*M*) = ∑λ, we get,

(5) $$\begin{array}{l} \sum \lambda \left(\nabla^{2}f\left(\theta \right)\right)=\underset{k}{\sum }\underset{i}{\sum }\nabla^{2}f\left(\theta_{ki}\right)\\ \;=-\frac{KI}{\sigma^{2}}+\underset{k}{\sum }\underset{i}{\sum }\frac{1}{\sigma^{2}}\left(\theta_{ki}-\mu \right)\\ \;+\underset{n}{\sum }\underset{k}{\sum }\underset{i}{\sum }w_{ki}\alpha e^{w_{ki}}\left(S_{k}\left(t\right)-\Theta \left\{h_{k}^{n}\right\}\right) \end{array}$$

It is obvious that ∑λ(∇^2^*f*(θ)) consists of three terms: $A=-\frac{KI}{\sigma^{2}}$, $B=\underset{k}{\sum }\underset{i}{\sum }\frac{1}{\sigma^{2}}\left(\theta_{ki}-\mu \right)$, $C=\underset{n}{\sum }\underset{k}{\sum }\underset{i}{\sum }w_{ki}\alpha e^{w_{ki}}\left(S_{k}\left(t\right)-\Theta \left\{h_{k}^{n}\right\}\right)$. We need to prove the following equality.

(6) $$\begin{array}{l} \sum \lambda \left(\nabla^{2}f\left(\theta \right)\right)=A+B+C>0 \end{array}$$

The proof is divided into three steps. Note that the first sentence of each step below is the conclusion we want to prove.

1. *B*≪*C*. Only when the noise $dW_{i}=N\left(0,N\alpha e^{w_{\text{ki}}}\right)dt$, we can derive that $B+\left(x_{i}^{n}-\alpha e^{w_{\text{ki}}}\right)C$ is a variant of the gradient. According to the zero gradient and STDP learning rule, $\frac{B}{C}\approx 0$, thereby B can be ignored.
2. C is positive. $\;C\approx N\left(\underset{i}{\sum }w_{\text{ki}}x_{i}-\underset{i}{\sum }w_{label,i}x_{i}\right)$ which represents the approximate potential difference of actual and expected neurons. When networks are trapped in saddle points, the neuron that releases spikes is not the one expected. Thus, the potential of actual neurons is higher than expected.
3. *A* + *B* + *C* > 0. A is a negative constant. When N is greater than a certain value, C is large enough so that *A* + *B* + *C* > 0 and the strict saddle property will be satisfied.

The theorem is therefore proven. That is to say, Theorem 1 guarantees noisy synaptic sampling networks satisfy the strict saddle condition, and hence noise will help escape from saddle points in Theorem 6 of the work (Ge et al., 2015). The detailed derivation appears in Appendix 1 (Supplementary Presentation 1) . It is worth noting that we found that the important step *C* represents the positive potential difference of actual and expected neurons, and thus the strict saddle condition can be satisfied as long as *C* is large enough. The realization of such an important step comes from introducing parameters $\pm N\alpha w_{ki}e^{w_{ki}}$ by proposed noise. In other words, noise helps spiking neural networks build appropriate Hessian construction, and optimization can utilize enriched curvature information of the node in the direction without ever calculating or storing the Hessian itself.

## 4. Biologically Interpretation for Proposed Noise

### 4.1. Origin From the Free-Energy Principle in Neuroscience

The proposed noise structure is inspired by the free-energy principle. It is generally believed in neuroscience that any adaptive system at equilibrium with its environment must minimize its free energy (Friston, 2010; Joffily and Coricelli, 2013; Apps and Tsakiris, 2014; Colombo and Wright, 2018). Free energy can be expressed as self-information plus a Kullback–Leibler divergence term in Equation (7), where $\tilde{s}$ is a sensation signal (Friston, 2010).

(7) $$\begin{array}{l} F=D\left(q\left(\vartheta |\mu \right)||p\left(\vartheta |\tilde{s}\right)\right)-lnp\left(\tilde{s}|m\right) \end{array}$$

Given the noise signals $\tilde{\varepsilon }$, the Kullback–Leibler divergence is the perceptual difference between the recognition density *q*(ϑ|μ) encoded by internal states μ and the posterior density $p\left(\vartheta |\tilde{\varepsilon }\right)$ of the causes ϑ. Self-information measures the error between the true and expected sensation. It is formally the negative log-probability of a noise outcome $\tilde{\varepsilon }$ given the generative model *m*, that is,$\;-lnp\left(\tilde{\varepsilon }|m\right)$. Equivalently, it is also expressed as the long-term average of the square error between the true and expected sensation. The divergence is always non-negative, and free energy is tightly bounded by surprise.

A number of cognition and perception studies show that brain system implicitly infers the cause (network parameters ϑ) in a Bayesian fashion (Ernst and Banks, 2002; Yuille and Kersten, 2006; Beck et al., 2008), the recognition density *q*(ϑ|μ) approximates the posterior density $p\left(\vartheta |\tilde{\varepsilon }\right)$. That is to say, Kullback–Leibler divergence approximates zero and free energy becomes surprise. Therefore, minimizing the free energy is also a process to minimize the error $E{\left(\varepsilon -\tilde{\varepsilon }\right)}^{2}$ between the true and expected noise outcome $\tilde{\varepsilon }$. Thereby, we obtain the optimal noise distribution as shown in Theorem 2.

****Theorem** 2 (free energy principle)**. *Suppose that a function *g*(*X*):*ℝ^*I*^ → ℝ^*K*^ *is a spiked-based winner-take-all neural network, given the output variable *z*^*n*^ = *k*, the value of input $x_{i}^{n}$ is from a Poisson distribution $POISSON\left(x_{i}^{n}|\alpha e^{w_{\text{ki}}}\right)$ where the mean is determined by the synaptic weight *w*_ki_ from input neuron i to network neuron k. If there is a noise distribution *p*(ε|θ) in Equation (8), such a self-organizing system can minimize its free energy of noise signals. Further, the optimal noise $\hat{\varepsilon }$ is obtained by the minimum mean square-error estimation $E\left(\varepsilon |\hat{\theta }\right)$*.

(8) $$\begin{array}{l} \varepsilon |\theta \sim N\left(0,N\alpha e^{w_{\text{ki}}}\right) \end{array}$$

The important step is to obtain the probability distribution *p*(ε|θ). By inducing input variables *x*, the unknown distribution *p*(ε|θ) will become the integration of easy distributions *p*(ε|*x*) and *p*(*x*|θ) in Equation (9). *p*(*x*|θ) is the normal distribution where both the mean and variance are $N\alpha e^{w_{\text{ki}}}$. It has been shown in the work by Habenschuss et al. (2013) and Kappel et al. (2015) that in the spiked-based WTA networks, one prominent motif of cortical microcircuits, *p*(*x*|θ) is the integration of *N* Poisson distribution with the mean $\alpha e^{w_{\text{ki}}}$, which can approximate normal distribution. The detailed proof appears in Appendix 2 (Supplementary Presentation 1) .

(9) $$\begin{array}{l} p\left(\varepsilon |\theta \right)=\int p\left(\varepsilon |x\right)p\left(x|\theta \right)\text{dx} \end{array}$$

In this section, we illustrate three points. First, the free energy principle helps verify the plausibility of the proposed noise. It is popular to use Brownian motion to describe continuous random fluctuations in spiking activities. In contrast, the standard deviation of our noise is *Nαe*^*w*^ while it is constant in traditional Brownian noise, which is an important difference to other similar noise models. According to free energy, only this type of noise, i.e., $N\left(0,N\alpha e^{w}\right)$ can be derived rather than standard Brownian noise or other forms. Therefore, it is strong evidence for the plausibility of the proposed noise theoretically. Second, the free energy principle improves biological relevance of our noise. In neuroscience, the free energy principle unifies different aspects of how the brain works, such as attention, synaptic plasticity, and neuronal coding. Satisfying the free energy principle complements evidence of the neurobiological existence of our proposed noise. Third, the origin of our proposed noise should be illustrated, and why we choose such type of noise, and not other types of Brownian noise in the strict-saddle condition, is answered. In fact, we derived the proposed noise initially inspired by the free energy principle. We then found that such noise helps spiking neural networks satisfy the strict-saddle condition.

### 4.2. Consistency With Biological Observations

**Figure 3:**
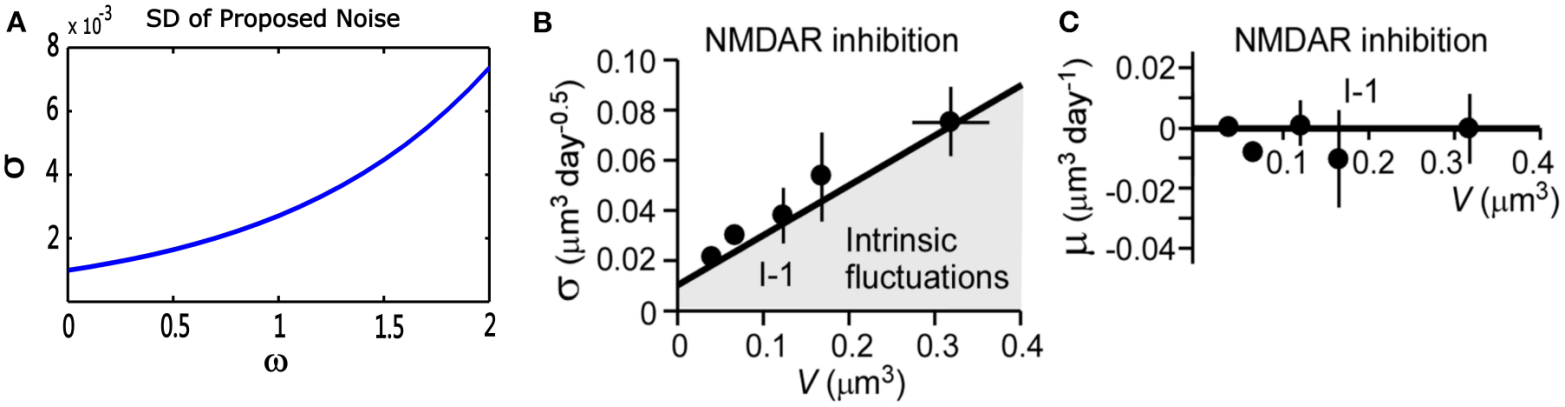
Quantitative analysis of noise from the proposed theory and physiological experiments in the absence of synaptic activity. **(A)** Standard deviation (σ) of proposed theoretical noise $N\left(0,N\alpha e^{w_{\text{ki}}}\right)$. When synaptic weight changes are relatively small, the standard deviation is roughly proportional to synaptic weights. **(B,C)** Standard deviation (σ) and mean (μ) of fluctuations in spine-head volume in the absence of activity-dependent plasticity in physiological experiments. Standard deviation (σ) is greater for larger spines. Further, (σ) has an approximately proportional relationship with spine-head volume. The mean change (μ) is around zero, which is consistent with that of our noise (cited in Figure 5 of the paper Nobuaki et al., 2009).

Many biological and biochemical stochastic processes affect the efficacy of a synaptic connection. Some are indirectly related, for example, NMDA receptors, PSD-95 in the mammalian postsynaptic density (PSD), which can affect the amplitude of postsynaptic potentials and the efficiency on clustering glutamate receptors (Bhalla and Iyengar, 1999; Gray et al., 2006; Coba et al., 2009; Ribrault et al., 2011). Some are directly related, such as the volume of spines at dendrites (Engert and Bonhoeffer, 1999; Matsuzaki et al., 2001; Zhong et al., 2005; Ho et al., 2011). It is popular to use Brownian motion W(t) to describe such random continuous fluctuations (Tuckwell, 1988; Cateau and Fukai, 2003; Câteau and Reyes, 2006; Nobuaki et al., 2009). Brownian motion W(t) is utilized in the Langevin equation as Equation (10) shown,

(10) $$\begin{array}{l} \frac{dV\left(t\right)}{dt}=\sigma \left(V\left(t\right)\right)\frac{dW\left(t\right)\left(t\right)}{dt}+\mu \left(V\left(t\right)\right) \end{array}$$

where *V*(*t*) represents a stochastic process with an average change (or drift) μ(*V*), and standard deviation σ(*V*), W(t) represents standard Brownian motion. Nobuaki et al. (2009) applied such a stochastic process in Equation (10) to the volume of spines *V*(*t*). They recorded the volumes of many individual spines of CA1 pyramidal neurons in a rat hippocampus. They found “intrinsic volume fluctuations” in the absence of synaptic activity. Figure 3 shows the corresponding quantitative analysis of fluctuations in spine-head volume in the absence of activity-dependent plasticity. It shows that average change μ(*V*) of intrinsic volume fluctuations is zero, and the standard deviation σ(*V*) is roughly proportional to the spine-head volume. It is likely because larger spines have a greater PSD area. Therefore, it will accumulate more AMPA-type glutamate receptors and more synaptic vesicles in the presynaptic terminal (Harris and Stevens, 1989; Nusser et al., 1998; Takumi et al., 1999; Harris et al., 2003; Knott et al., 2006). In our noise structure $N(0,N\alpha e^{w})$, σ is also greater for larger spines. It is consistent with this important observation of spine dynamics, and further, it may be a more plausible model to describe intrinsic fluctuations.

## 5. Extension to Multi-Layer Noisy Spiking Neural Networks

**Figure 4:**
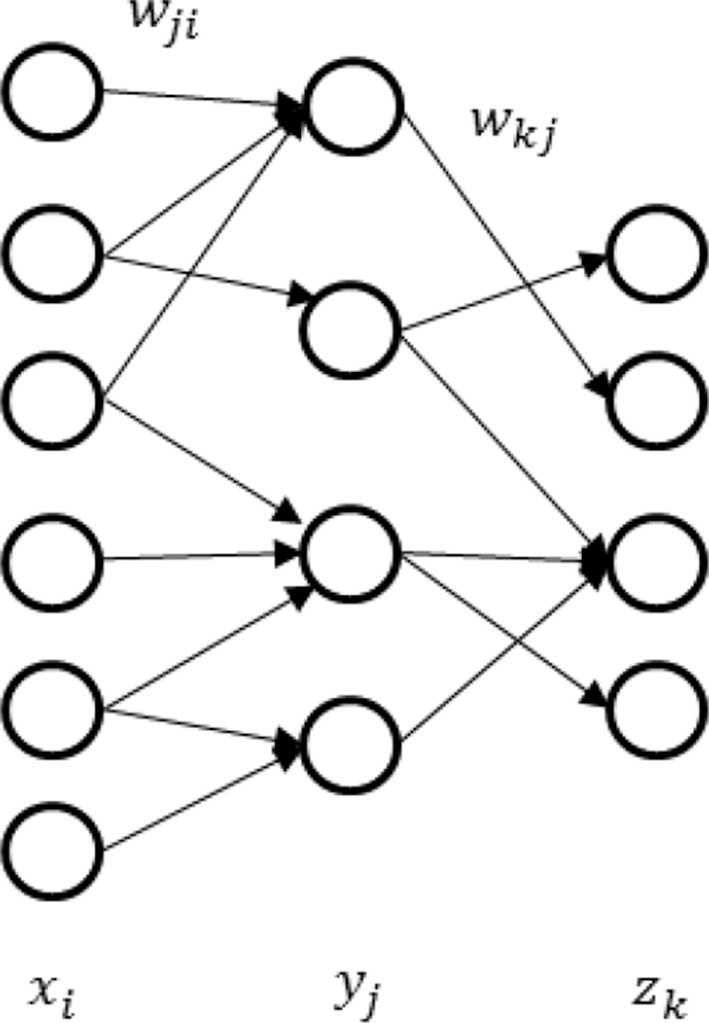
Three-layer neural networks diagram.

In this section, we will demonstrate one computational application of our noise model: realization of multi-layer spiking neural network for better representations. These characteristics will shed new light on machine learning. It is generally believed that deep neural networks can learn representations better than the two-layer network and are more extensively applied in various scenarios (LeCun et al., 2015). It is therefore significant to generalize the depth of noisy spiking neural networks. As an example, we derive a back-propagation algorithm for synaptic sampling on the three-layer network in Figure 4. The derivation for deeper networks is similar.

The prior probability remains the same, which reflects the structural constraints and rules. The likelihood function is still the form of cross-entropy, which reflects the class recognition probabilities. The difference is the posterior probability *p*(*z*^*n*^ = *k*|*x*^*n*^, *w*) becomes the product of Poisson distributions of both the first and second layers. The likelihood function in Table 1 becomes,

(11) $$\begin{array}{l} \log p_{N}\left(J|\theta \right)=\overset{N}{\underset{n=1}{\sum }}\overset{K}{\underset{k=1}{\sum }}\Theta \left\{h_{k}^{n}\right\}\log p\left(z^{n}=k|x^{n},w\right)\\ =\overset{N}{\underset{n=1}{\sum }}\overset{K}{\underset{k=1}{\sum }}\Theta \left\{h_{k}^{n}\right\}\log\\ \frac{\prod_{i}\sum_{j}\text{POISSON}\left(x_{i}^{n}|\alpha e^{w_{\text{ji}}}\right)POISSON\left(y_{j}^{n}|\alpha e^{w_{\text{kj}}}\right)}{\sum_{k}\prod_{i}\sum_{j}\text{POISSON}\left(x_{i}^{n}|\alpha e^{w_{\text{ji}}}\right)POISSON\left(y_{j}^{n}|\alpha e^{w_{\text{kj}}}\right)} \end{array}$$

The main difficulty is how to get the gradient in the first layer. According to the generalized delta rule, derivative results from the product of two parts: one part represents the change in likelihood function relative to the change of net inputs, and one part reflects the change of net inputs relative to a small change of weight. Thus, we get,

(12) $$\begin{array}{l} \frac{\partial \log p_{N}\left(J|\theta \right)}{\partial w_{\text{ji}}}=\frac{\partial \log p_{N}\left(J|\theta \right)}{\partial y_{j}}\frac{\partial y_{j}}{\partial w_{\text{ji}}} \end{array}$$

where *y*_*j*_ is net inputs for the second layer. For the second factor, as the firing rate of stochastic spike response neurons depends exponentially on the membrane voltage (Jolivet et al., 2006; Mensi, 2011), we derive that,

(13) $$\begin{array}{l} \frac{\partial y_{j}}{\partial w_{\text{ji}}}\approx x_{i} \end{array}$$

For the first factor, it can be implemented by propagating gradient information backward through the last layer. According to the chain rule, it can also be written as the product of two parts, as shown in (Equation 14).

(14) $$\begin{array}{l} \frac{\partial \log p_{N}\left(J|\theta \right)}{\partial y_{j}}=\underset{k}{\sum }\frac{\partial logp_{N}\left(J|\theta \right)}{\partial w_{kj}}\frac{\partial w_{\text{kj}}}{\partial y_{j}} \end{array}$$

One part is the gradient of the last layer, and another part is simply the deviation of mean function *E*(*y*_*j*_) determined by the synaptic weight *w*_kj_ from input neuron *j* to neuron *k*. In this case, substituting Equations (13) and (14) to Equation (12), we finally get the gradient information of the first layer.

Given the L-layer noisy spiking neural networks, each layer computes a function $X^{l}=g_{l}\left(X^{l-1},W^{l}\right)$, where *X*^*l*^ is the output of the *l*_*th*_ layer, *X*^*l*−1^is the input of the *l*_*th*_ layer and *W*^*l*^ is the vector of adjustable parameters between the (*l* − 1)_*th*_ and the *l*_*th*_ layer. Note the vector *X*^1^ in the first layer is the input sample. The learning rule for L-layer spiking neural networks can be concluded as follow. The detailed derivation appears in Appendix 3 (Supplementary Presentation 1) .

(15) $$\begin{array}{l} d\theta_{kj}^{L}=b(\frac{1}{\sigma^{2}}\left(\mu -\theta_{\text{kj}}^{L}\right)+\sum_{n=1}^{N}(w_{kj}^{L}\left(x_{j}^{n,L-1}-\alpha e^{w_{\text{kj}}^{L}}\right)(\Theta \{h_{k}^{n}\}\\ -S_{k}^{L}\left(t\right)))dt+bdW_{\text{kj}}^{L} \end{array}$$

(16) $$\begin{array}{l} d\theta_{\text{ji}}^{l-1}=\beta \alpha \sum_{n=1}^{N}x_{i}^{n,l-1}\left(d\theta_{\text{kj}}^{l}+d\theta_{\text{mj}}^{l}\right)\;(2\leq l\leq L) \end{array}$$

where $d\theta_{\text{kj}}^{l}$ represents change in parameters corresponding to the neuron *k* which releases a spike and $d\theta_{\text{mj}}^{l}$ represents change in parameters corresponding to the desired neuron *m*.

## 6. Simulation Results

We run simulations for synaptic sampling with/without noise applied to 10-categories of classification. We test the proposed model on three datasets from three aspects: (1) application accuracy; (2) neuron spike responses; (3) reduction rates for trapping in saddle points.

### 6.1. Sensory Environment

#### 6.1.1. Synthesis Dataset

**Figure 5:**
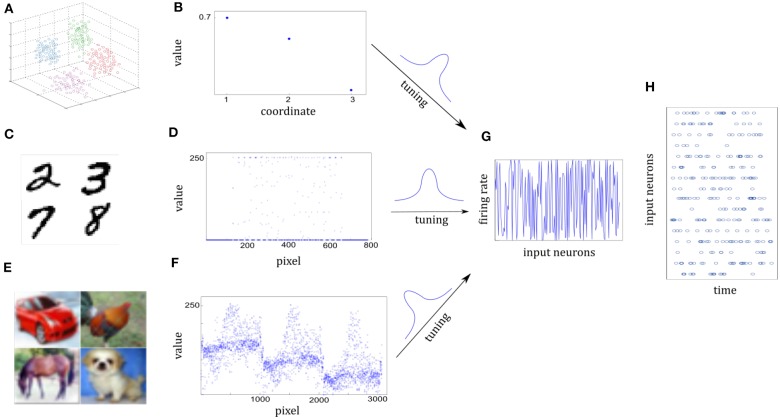
Illustration of sensory information tuning to network inputs. **(A,B)** Examples of 3D points from the Synthesis dataset. Different classes of sensory information are represented by different clusters of 3D points, each illustrated by different colors. Three coordinate values of one 3D point are shown in **(B)**. They will be tuned by the input neurons. **(C,D)** Examples of digits from MNIST dataset. Handwritten digit is a 28 × 28 pixel image with the gray value. Seven hundred and eighty-four pixel values of one image are shown in **(D)**. **(E,F)** Examples of images from CIFAR-10 dataset. Each image is a 32 × 32 color image in the RGB format. 3,072 pixel values of one image are shown in **(F)**. **(G)** Firing rates of input neurons after tuning the normalized sensory information. Firing rates are distributed almost uniformly in spite of different sensory representations in **(B,D,F)**. **(H)** Spike trains of some of the input neurons. Given each example, firing rates are kept fixed and Poisson spike trains are drawn for the 200-ms duration of the input presentation.

We use a cluster of points in 3D space to represent one sensory experience for visualization. The center of a cluster is the mean of the Gaussian, which is independently drawn from N(0.5,0.2). The covariance matrix of the cluster is randomly given by 0.04*I*+0.01ξ, where *I* is the 3-dimensional identity matrix and the element of ξ is randomly drawn from N(0,1). Figure 5A shows some clusters of points in 3D space. Each cluster represents one class. Different cluster are described by different color. Figure 5B shows three-dimensional coordinates. For 10-categories classification, 10 clusters will be generated equally. We generate a sample by randomly selecting one Gaussian cluster and then get a sample position from the corresponding distribution.

#### 6.1.2. MNIST Dataset

It composes of 10 handwritten Arabic numbers from 0 to 9, which has a training set of 60,000 examples, and a test set of 10,000 examples. It is a good compromise between real-world data test and easy preprocessing and formatting. Each example is a 28 × 28 pixel image with the gray-scale value. In preprocessing the pixel values are normalized to interval [0, 1]. Images in Figure 5C are drawn from MNIST dataset. Figure 5D shows the 784 pixel values of one 28 × 28 grayscale image.

#### 6.1.3. CIFAR-10 Dataset

It consists of 60,000 color images in 10 classes, which has a training set of 50,000 examples, and a test set of 10,000 examples. The 10 classes are completely mutually exclusive, such as airplane, bird, and cat. Each example is a 32 × 32 color image in the RGB format. In preprocessing the pixel values are normalized to interval [0, 1]. In Figure 5F, it consists of three similar parts due to RGB format.

#### 6.1.4. Network Inputs

Both the sample positions in the Synthesis dataset and real-world images from the MNIST or CIFAR-10 dataset are represented by the spatiotemporal spike trains. For the Synthesis dataset, the input layer is 1,000 neurons for one 3D point while for real-world datasets, each pixel of one image is represented by a single input neuron. Input neurons have different Gaussian tuning curves. According to tuning curves, they tune the sample position or normalized pixel values to corresponding firing rates. In addition, the 5 Hz background noise is added. Although raw sensory values of three datasets are differently distributed in Figures 5B,D,F, corresponding firing rates are scattered over almost the entire probability space after tuning in Figure 5G. As a result, Poisson spike trains of each input neuron are drawn with duration of 200 ms in Figure 5H.

#### 6.1.5. Settings

In all simulations, we set *N* = 1, 000, α = *e*^−6^, and *b* = 10^−5^ or *b* = 10^−4^. Initial synaptic parameters are drawn from the prior distribution *p*_*S*_(**θ**). We adopt the same configuration about the offset θ_0_ and actual weights ${\hat{w}}_{\text{ki}}$ with Kappel et al. (2015)

The purpose of our paper is to propose one appropriate computational hypothesis about whether biologically inherent noise benefits neural systems and how it occurs precisely. To test this hypothesis, we chose the biologically appropriate neural model: synaptic sampling. On the one hand, the inherent noise is described in variables in synaptic sampling and hence it easy to capture the details of noise biophysics and dynamics. The advantage of noise can be analyzed based on mathematical tractability. On the other hand, synaptic sampling is a biologically appropriate neural system since it simulates some aspects of realistic neural systems, such as neuron topology, neuron type (e.g., excitatory, inhibitory), and Spike Timing Dependent Plasticity (STDP) learning rule, spatial and temporal effect of spike signals. The goal of our paper has been achieved when learning performance is better in the presence of noise than in its absence in the synaptic sampling experiments. Although it may perform better with further hand-tuning, it is beyond the scope of this paper. As far as we know, we first realize the supervised learning in the synaptic sampling networks. Apart from the stochastic term, the parameter configuration in synaptic sampling without noise is the same as that with noise. Therefore, synaptic sampling without noise is representative of the basic model.

### 6.2. Verification on the Two-Layer Networks

**Figure 6:**
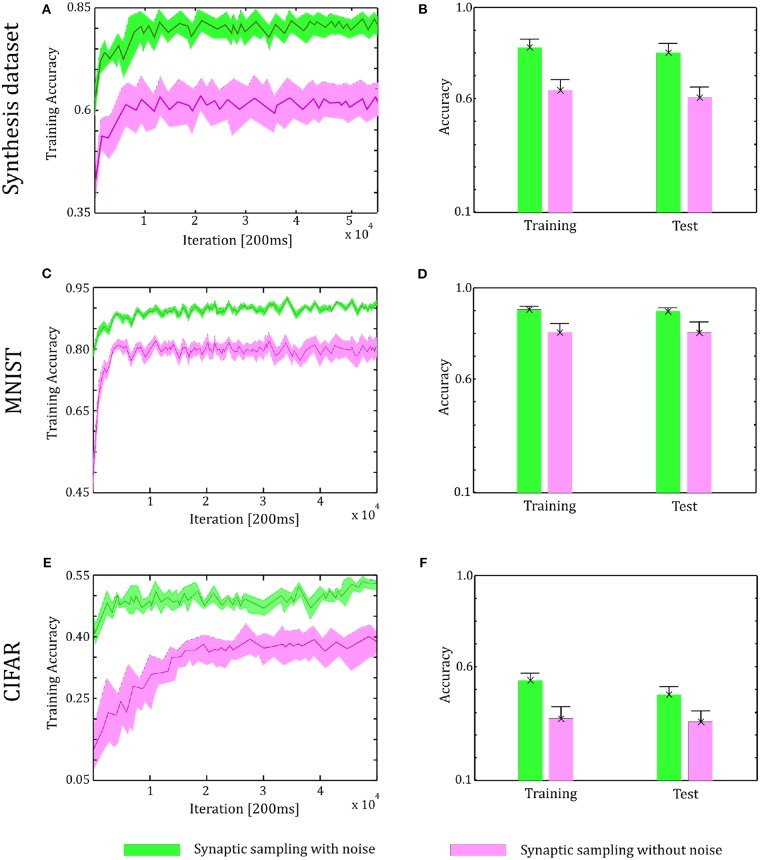
Performance of the two-layer networks with and without internal noise. **(A)** Learning curves of synaptic sampling with/without noise on the training set for the Synthesis dataset. Mean values over 10 runs are shown, shaded area indicates STD. **(B)** Accuracy comparison in the learning and test phase for Synthesis dataset (averaged over 10 runs). Error bars indicate STD. **(C,D)** In the MNIST dataset, the performance with internal noise maintains better than that without noise throughout the whole learning course. **(E,F)** In the CIFAR-10 dataset, the performance with internal noise maintains better than that without noise throughout the whole learning course.

Through the tuning curves of input neurons, 200 ms spike patterns were communicated to synaptic sampling networks for each sample. According to Equation (3) and spike-based update scheme, the sensory experiences were presented sequentially, and all synapses were updated sequentially. The final predicted label is the neuron which fires most between the 10 output neurons. We repeat the simulation 10 runs and the accuracy is averaged over 10 runs. For the Synthesis dataset, we present 14,400 samples to 1,000 input neurons for 2.4 h. For the MNIST dataset, we present 60,000 samples to 784 input neurons. For the CIFAR-10 dataset, we present 50,000 samples to 3,072 input neurons. As shown in Figure 6, in the learning and test phase, the accuracy of synaptic sampling is almost 20% higher than that without noise for the Synthesis dataset. The gap of accuracy with/without noise is around 15% for the CIFAR-10 dataset and around 10% for the MNIST dataset. The accuracy of synaptic sampling without noise in these datasets is, respectively around 80% (MNIST), 39% (CIFAR-10), 60% (Synthesis dataset). It shows the number of bad saddle points in the spiking neural network is relatively small given the MNIST dataset. Therefore, synaptic sampling without noise also obtains satisfactory performance, and adding noise obtains the least increase in accuracy compared to other datasets. On the other hand, the CIFAR-10 dataset is the most challenging among three datasets due to the larger scale and more complex representation as shown in Figure 5. It indicates that there will be a large number of bad saddle points making it difficult to achieve a significantly better performance. As a result, the accuracy is only improved from 40% (without noise) to 55% (with noise). For the three datasets, we found that synaptic sampling with/without noise tends to converge at 10,000th iteration. Although the speed of convergence in synaptic sampling with/without noise is similar, synaptic sampling with noise is faster than that without noise, especially in the CIFAR-10 dataset. It is likely because spiking neural networks with noise build more appropriate Hessian construction and utilize enriched curvature information of the node in the direction. As shown in Figures 6B,D,F, the standard deviation of synaptic sampling with noise is slightly smaller in spite of additional fluctuations. That is to say, the precision of the optimization performance does not become worse with the effect of noise, which is different from the general idea that noise will lose precision. In addition, the accuracy in the test phase is similar to that in the learning phase. It shows that noise prevents spiking neural networks from overfitting in spite of increasing learning accuracy.

**Figure 7:**
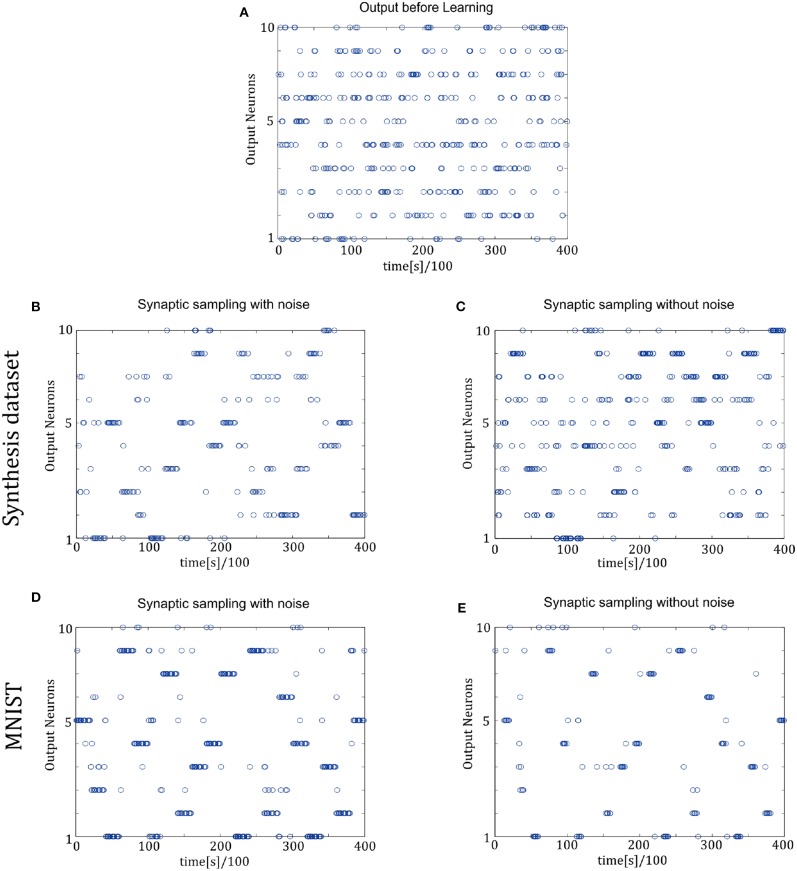
Spiking activity of the 10 output neurons in the two-layer networks with/without internal noise motifs. We present 20 samples during a 4 s epoch for one of the 10 simulations. Ten output neurons in the WTA circuits represent the binary random variables in the supervised classification learning. **(A)** Firing responses of the 10 output neurons before learning. **(B,C)** Firing responses of the output neurons after learning with/without noise for Synthesis dataset. Sparsification of firing of output neurons occurs obviously after learning with noise. **(D,E)** Same for MNIST dataset. The corresponding labels of 20 samples are [6, 3, 1, 9, 5, 1, 8, 2, 4, 5, 8, 1, 9, 2, 7, 5, 1, 4, 2, 6]. The network enters and remains in different network states (indicated by different positions of grouped spikes), corresponding to different predicted class in the supervised learning. The tight match between labels and preference neurons suggests that the generated internal model encodes the MNIST representation efficiently.

Figure 7 shows the spike trains of 10 readout neurons in the time course of learning. The ordinate displays 10 readout neurons. The abscissa displays time. One point (*t, x*) represents that at the time *t*, neuron *x* releases one spike. In Figures 7D,E, it shows the 400-ms learning process from the 57,000th example to the 57,020th example. The corresponding label is [6, 3, 1, 9, 5, 1, 8, 2, 4, 5, 8, 1, 9, 2, 7, 5, 1, 4, 2, 6]. In the learning process, spikes are scattered almost equally among 10 readout neurons initially and hence responses are unspecific to different inputs in Figure 7A. However, finally spikes are grouped at the positions from the 6th, 3rd, 1st, 9th, 5th, 1st, 8th, 2nd, 4th, 5th, 8th, 1st, 9th, 2nd, 7th, 5th, 1st, 4th, 2nd, 6th neurons in Figure 7D, which is the same as label order. Therefore, responses have become preferences for different inputs. Synaptic sampling with noise obtains the best learning results of this task, and the corresponding spike responses have the most obvious preference pattern. In Figure 7E, less spikes are grouped at the positions which is the same as label order. Because synaptic sampling without noise learns this task less accurately. In Figure 7C, preference pattern is not very obvious since synaptic sampling without noise cannot learn this task accurately for Synthesis dataset.

Here, we need to discuss one similar work for additional verification of noise effects. Based on synaptic sampling, Kappel et al. (2018) presented a framework to maintain the stable computational power in spite of stochastic spine dynamics. They proposed that the functional role of noise is the compensation for network perturbations and hence noise can help network maintain the stable computational power. They also conducted the experiments about different temperature on learning performance, where the strength of stochasticity can be scaled by the temperature. The results show that good performance was achieved for a range of temperature values, and too low (such as without noise) or too high temperature impaired learning, as shown in the Figures 5D,G of their paper. To some extent, it is of evidence that synaptic sampling with noise indeed obtains better performance. However, the difference with our work is outlined as three points.

First, the type of noise is different. They use the standard Brownian noise and the strength of stochasticity should be scaled by the temperature. Therefore, it is an issue to adjust the strength of noise. In contrast, the strength of our noise can be adapted automatically based on the synaptic weight. Second, the perspective from why the noise works is different. They mainly focus on the realization of stable computational function instead of noise mechanism, and therefore provide a short analogy using simulated annealing. However, we analyze the noise theoretically from the view that noise helps optimization escape from saddle points. Third, the application is different. Their model is in the context of reinforcement learning, i.e., lever movement, while our model is in the context of supervised learning, i.e., classification.

### 6.3. Verification on the Three-Layer Networks

In the simulations of three-layer networks, we present 43,200 samples to networks for the Synthesis dataset and 60,000 samples for the MNIST dataset. Other configurations are the same as two-layer networks. The number of neurons in the hidden layer is 500. We repeat the simulation 10 runs and the accuracy is averaged over 10 runs.

**Figure 8:**
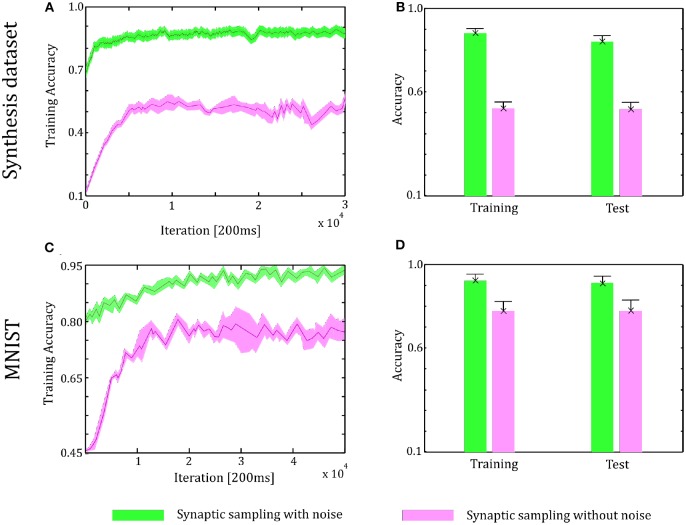
Performance of the three-layer networks with and without internal noise. **(A)** Learning curves of synaptic sampling with/without noise on the training set for Synthesis dataset. Mean values over 10 runs are shown, shaded area indicates STD. **(B)** Accuracy comparison in the learning and test phase for the Synthesis dataset (averaged over 10 runs). Error bars indicate STD. **(C,D)** In the MNIST dataset, the performance with internal noise is better than that without noise throughout the whole learning course.

As shown in Figure 8, in the learning and test phase, the accuracy of synaptic sampling is almost 30% higher than that without noise for the Synthesis dataset and around 15% for the MNIST dataset. Compared with the two-layer network in Figure 6, when the number of layers increase, the performance becomes better, i.e., the accuracy is improved from 80% in two-layer networks to 88% in three-layer network for the Synthesis dataset. It is because deeper networks have the potential for more complex representation. However, the accuracy of synaptic sampling without noise has not improved in spite of the increasing number of layers as the number of saddle points increases exponentially. As shown in Figures 8B,D, likely, the standard deviation of synaptic sampling with noise is slightly smaller in spite of additional fluctuations on the three-layer networks. Therefore, noise improving optimization without losing precision can also be applied to three-layer spiking neural networks. In addition, the accuracy in the test phase is similar to that in the learning phase. It shows that noise prevents spiking neural networks from overfitting, regardless of the increasing number of layers. In addition, the speed of convergence in synaptic sampling slows down in three-layer networks, especially without noise, compared to Figures 6C, 8C. The increasing number of layers leads to the increasing number of saddle points and hence it is more difficult for networks to escape from saddle points without noise.

**Figure 9:**
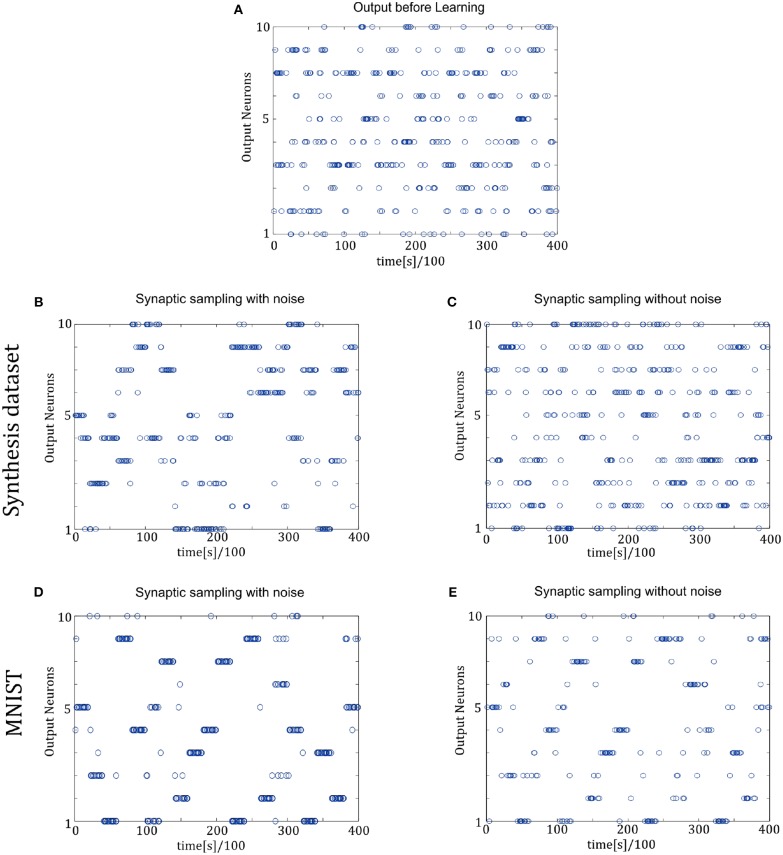
Spiking activity of the 10 output neurons in the three-layer networks with/without internal noise motifs. We present 20 samples during a 4 s epoch for one of the ten simulations. **(A)** Firing response of the 10 output neurons before learning. **(B,C)** Firing response of the output neurons after learning with/without noise for Synthesis dataset. Sparsification of firing of output neurons occurs obviously after learning with noise. **(D,E)** Same for MNIST dataset. The corresponding labels of 20 samples are [6, 3, 1, 9, 5, 1, 8, 2, 4, 5, 8, 1, 9, 2, 7, 5, 1, 4, 2, 6].

In Figure 9, the spike responses are similar to that of the two-layer networks. For the Synthesis dataset, initial responses are unspecific to different inputs. After learning, synaptic sampling with noise learns the best results of this task, and the corresponding spike responses have the most obvious preference pattern. Synaptic sampling without noise cannot learn this task accurately, and the preference pattern is not very obvious in Figure 8C. For the MNIST dataset, Figures 8D,E show the 400-ms learning process from the 57,000th example to the 57,020th example. The corresponding labels are [6, 3, 1, 9, 5, 1, 8, 2, 4, 5, 8, 1, 9, 2, 7, 5, 1, 4, 2, 6]. After learning, more spikes are grouped at the position as indicated by the label number, compared to synaptic sampling without noise. Therefore, synaptic sampling learns the task better and spike responses have more obvious preference‘pattern.

### 6.4. Verification for Escaping From the Saddle Points

We try to clarify that the gain in performance in the experiments is due to overcoming the saddle point problem. Although it is difficult to calculate the total number of saddle points and strict-saddle points, it is easy to calculate the total number of strict-saddle points which satisfies Equation (6) given the inputs and network weights. Note that strict-saddle points which satisfies Equation (6) is the subset of strict-saddle points, and hence it is plausible to test the existence of escaping from the saddle points with noise. In Equation (5), given different inputs, the Hessian property of a single weight may be different. Therefore, the main configuration of the strict-saddle point depends on the inputs and network weights. Based on the experiments in section 6.2, we choose the 30 weights at the convergence stage and 1,000 inputs for a total 30,000 samples. If Equation (6) is satisfied given one sample, the corresponding weights belong to a strict-saddle point. In this way, we count the number of strict-saddle points in the spiking neural networks with/without noise, denoted as *S*_1_ and *S*_2_, respectively. Then, we calculate the reduction rate of strict-saddle points. To some extent, the reduction rate reflects how effectively noise prevents the learning algorithm from trapping in the saddle points. The reduction rate is calculated according to Equation (17).

(17) $$\begin{array}{l} Reduction\;Rate=\frac{S_{2}-S_{1}}{S_{2}} \end{array}$$

The results are shown in Table 2. It obvious that the strict saddle points with noise has been reduced greatly for three datasets, which shows that noise helps optimization escape from many saddle points. In addition, we sort the datasets according to the reduction rate in descending order, that is, the Synthesis dataset >CIFAR-10 >MNIST. According to the accuracy gain in descending order, the list is, Synthesis dataset >CIFAR-10 >MNIST. Therefore, the above two sort lists show that when the reduction rate is greater, performance improves, and noise helps optimization overcome the saddle point problem more efficiently. Such positive correlation indicates that the gain in performance is due to overcoming the saddle point problem as suggested in our manuscript. The number of strict saddle points in the MNIST dataset is the smallest (i.e., 489) and hence synaptic sampling without noise can also obtain the satisfactory performance (~80%). For the CIFAR-10 dataset, although the reduction rate is not small (68.64%), the number of strict saddle points is still large in contrast (i.e., 6,157) due to the larger scale and more complex representation. Therefore, synaptic sampling with noise only achieves around 54% accuracy.

**Table 2:** Reduction results of strict-saddle points and the corresponding gain in accuracy.

**Dataset**	**Number of strict saddle points**		**Reduction rate (%)**	**Accuracy (%)**		**Accuracy gain (%)**
	**With noise (*S*_1_)**	**Without noise (*S*_2_)**		**With noise**	**Without noise**	
Synthesis dataset	711	4,157	82.89	~82	~60	~22
MNIST	489	1,167	58.06	~90	~80	~10
CIFAR-10	6,157	19,632	68.64	~54	~39	~15

## 7. Conclusion and Discussion

In this work, we introduce one biologically plausible noise structure, which is different from the traditional Gaussian noise, and investigate the noise mechanism on the brain computation theoretically. We applied the proposed model to 10-categories classification problem to demonstrate the learning ability of noisy spiking neural networks and compared with networks without noise. Simulation results show that noisy spiking neural networks have higher learning accuracy, and spike responses had a more obvious preference pattern for random spike train inputs. Further, three-layer noisy spiking neural networks have better learning abilities compared with two-layer networks. Our contributions are three fold.

From the perspective of optimization, we propose that one of the essential roles of noise is to improve optimization in the brain computations and brain plasticity. Noisy spiking networks for which the synaptic weights affect the noise variance satisfy strict saddle conditions. In other words, proposed noise brings more descent directions and Hessian information of networks by changing the curvature of the landscape in the network parameter space, especially in the neighborhood near saddle points. In this case, gradient descent with noise will escape from bad saddle points leading to efficient optimization.

From the perspective of biology, we give a theoretical conjecture about the form of noise in the brain. The difference between our noise and traditional Gaussian noise is a positive correlation with a dendritic spine size. There are two biological proofs on such a plausible structure. First, we prove that the probability distribution of proposed noise satisfies the minimum mean square-error estimation based on the free energy principle in neuroscience. Second, it has been observed that larger spines show the most diverse changes in CA1 pyramidal neurons *in vivo* experiments, which is consistent with our noise structure.

From the perspective of the application, our noisy spiking neural networks can be extended to multi-layer networks based on the back-propagation algorithm. Deep learning has excellent abilities in learning complex representations due to its deep network structure. We hope that multi-layer noisy spiking neural networks can serve as a first step toward the realization of more powerful computation.

There are still some related open problems. First, the proposed noise is associated with the number of samples. It is worthwhile to study whether it can be automatically adaptive to some application that is sensitive to the sample size. For example, it has been tested by many studies in machine learning that on-line learning is better in large scale problems, while batch learning is better in small scale problems (Bottou and Bousquet, 2008; Mairal et al., 2009; Hoffman et al., 2010; Lin, 2010; Welling and Teh, 2011; Hardt et al., 2015). We hope the adaptive structure to the sample size in our noise will take effect on the robustness of sample size in artificial intelligence. Another important problem is whether there is a close relationship between proposed noise and similar techniques of adding noise in machine learning such as drop out, on-line learning. In future works, we will try to connect artificial and biological spiking neural networks further by studying the above two problems.

## Data Availability Statement

The datasets generated for this study are available on request to the corresponding author.

## Author Contributions

YF, ZY, and FC made important contributions to the conception of theory, design, and drafting of the manuscripts. YF designed and performed the simulations. All authors have approved publications in their current form. All authors agree to be responsible for all aspects of the work to ensure proper investigation and resolution of issues related to the accuracy or completeness of any part of the work.

## Conflict of Interest

The authors declare that the research was conducted in the absence of any commercial or financial relationships that could be construed as a potential conflict of interest.
