# Supplementary material for: Noise Helps Optimization Escape From Saddle Points in the Synaptic Plasticity
Source: Front Neurosci. 2020 Apr 29;14:343. doi: 10.3389/fnins.2020.00343 (PMC7201302; doi:10.3389/fnins.2020.00343)
Supplement: Supplementary file 1 [file Presentation_1.pdf]

Supplementary Material

1 PROOF OF THEOREM 1

The sufficient condition of strict saddle property is $\lambda_{\max}\left(\nabla^2 f(\theta)\right) > 0$. It is convenient to use a more strong condition, i.e., $\sum \lambda(\nabla^2 f(\theta)) > 0$. According to the equation about trace of $n \times n$ matrix A: $tr(A) = \lambda_1 + ... + \lambda_n$, we concentrate on the diagonal elements of the Hessian matrix. According to the chain rule, we convert the derivative of θ to w. We get that $\nabla f(\theta) = 0 \Leftrightarrow \nabla f(w) = 0$ and $\sum_k \sum_i \nabla^2 f(\theta_{ki}) \geq 0 \Leftrightarrow \sum_k \sum_i (\nabla^2 f(w_{ki})) w_{ki}^2 \geq 0$.

In the synaptic sampling neural networks, the objective function $f(\theta)$ defined by $logp^*(\boldsymbol{\theta}|J)$, where

$$\log p_S(\theta) = -\frac{(x-\mu)^2}{2\sigma^2} - \log\sqrt{2\pi}\sigma \tag{S1}$$

$$\log p_N(J|\theta) = \sum_{n=1}^{N}\sum_{k=1}^{K} \Theta\{h_k^n\} logp\left(z^n = k | x^n, w\right) \tag{S2}$$

Assuming that $A_{\text{ki}} = POISSON\left(x_i^n | \alpha e^{w_{\text{ki}}}\right) = \frac{(\alpha e^{w_{\text{ki}}})^{x_i^n} e^{-\alpha e^{w_{\text{ki}}}}}{x_i^n!}$, we get $p_N\left(x^n | z^n = k, w\right) = \prod_j \text{POISSON}\left(x_j^n \middle| \alpha e^{w_{\text{kj}}}\right) = \prod_j A_{\text{kj}}$. Therefore,

$$\frac{\partial(\log p\left(x|k,w\right))}{\partial w_{\text{ki}}} = \frac{1}{\prod_j A_{\text{kj}}} \prod_j A_{\text{kj}}\left(x_i^n - \alpha e^{w_{\text{ki}}}\right) = x_i^n - \alpha e^{w_{\text{ki}}} \tag{S3}$$

$$\begin{aligned}\frac{\partial\left(\log \Sigma_q p\left(x|q,w\right)\right)}{\partial w_{\text{ki}}} &= \frac{\prod_j A_{\text{kj}}\left(x_i^n - \alpha e^{w_{\text{ki}}}\right)}{\Sigma_q p\left(x|q,w\right)} = \frac{p\left(x|k,w\right)}{\Sigma_q p\left(x|q,w\right)}\left(x_i^n - \alpha e^{w_{\text{ki}}}\right) \\ &= p\left(k|\mathbf{x},\mathbf{W}\right)\left(x_i^n - \alpha e^{w_{\text{ki}}}\right)\end{aligned} \tag{S4}$$

We further get

$$\begin{aligned}\frac{\partial \log p_N(J|\theta)}{\partial w_{\text{ki}}} &= \frac{\partial \sum_{n=1}^{N}\sum_{k=1}^{K} \Theta\{h_k^n\} log\frac{p(x^n|z^n=k,w)}{\sum_q p(x^n|z^n=q,w)}}{\partial w_{\text{ki}}} \\ &= \sum_{n=1}^{N} \Theta\{h_k^n\} \frac{\partial(\log p\left(x|k,w\right) - \log \Sigma_k p\left(x|k,w\right))}{\partial w_{\text{ki}}} \\ &= \sum_{n=1}^{N} \Theta\{h_k^n\}\left(x_i^n - \alpha e^{w_{\text{ki}}}\right)\left(1 - p\left(k|\mathbf{x},\mathbf{W}\right)\right) \\ &\approx \sum_{n=1}^{N}\left(x_i^n - \alpha e^{w_{\text{ki}}}\right)\left(\Theta\{h_k^n\} - S_k(t)\right)\end{aligned} \tag{S5}$$

$$\frac{\partial^2 \log p_N(J|\theta)}{\partial^2 w_{\mathrm{ki}}} \tag{S6}$$
$$= \sum_{n=1}^{N} \frac{\partial \left(x_i^n - \alpha e^{w_{\mathrm{ki}}}\right) \big(\Theta\{h_k^n\} - p\left(k|\mathbf{x}, \mathbf{W}\right)\big)}{\partial w_{\mathrm{ki}}}$$
$$= \sum_{n=1}^{N} \left\{-\alpha e^{w_{\mathrm{ki}}} \left(\Theta\{h_k^n\} - p\left(k|\mathbf{x}, \mathbf{W}\right)\right) - \left(x_i^n - \alpha e^{w_{\mathrm{ki}}}\right)^2 p\left(k|\mathbf{x}, \mathbf{W}\right) \left(1 - p\left(k|\mathbf{x}, \mathbf{W}\right)\right)\right\}$$

In the spike-based Winner-Take-All networks, give the input x^n and the firing rate of neuron z_k is proportional to the posterior distribution $p\left(h^n = k|\boldsymbol{x^n}, \boldsymbol{w}\right)$. In simulation, the network generates spike trains $S_k(t)$ with such rate $\rho_k(t)$ of neuron z_k. According to $\frac{\partial^2 \log p_N}{\partial^2 \theta_{\mathrm{ki}}} = \left[\frac{\partial \log p_N}{\partial w_{\mathrm{ki}}} + w_{\mathrm{ki}} \frac{\partial^2 \log p_N}{\partial^2 w_{\mathrm{ki}}}\right] w_{\mathrm{ki}}$,

$$\frac{\partial^2 \log p_N(J|\theta)}{\partial^2 \theta_{\mathrm{ki}}} = \sum_{n=1}^{N} w_{\mathrm{ki}} \left(x_i^n - \alpha e^{w_{\mathrm{ki}}}\right) \big(\Theta\{h_k^n\} - S_k\left(t\right)\big) \tag{S7}$$
$$+ \sum_{n=1}^{N} w_{\mathrm{ki}}^2 \{-\alpha e^{w_{\mathrm{ki}}} \big(\Theta\{h_k^n\} - S_k\left(t\right)\big) - \left(x_i^n - \alpha e^{w_{\mathrm{ki}}}\right)^2 S_k\left(t\right) \left(1 - S_k\left(t\right)\right)\}$$

Due to the Gauss property $p\left\{|x - \mu| < \sigma\right\} = 0.6826$ and $\frac{w_{ki} e^{w_{ki}}}{\sqrt{e^{w_{ki}}}}\Big|_{w_{ki} \to 0} = 0$, it is plausible to refer to $\left(\sum_n \alpha w_{\mathrm{ki}} e^{w_{\mathrm{ki}}} \big(S_k\left(t\right) - \Theta\{h_k^n\}\big)\right) dt$ as the general characteristic of the noise distribution $d\mathcal{W}_{\mathrm{ki}}$. We further get the Hessian information with the noise,

$$\begin{aligned}
\nabla^2 \widetilde{f}\left(\theta_{\mathrm{ki}}\right) &= \frac{\partial^2 \log p_S\left(\theta\right)}{\partial^2 \theta_{\mathrm{ki}}} + \frac{\partial^2 \log p_N(J|\theta)}{\partial^2 \theta_{\mathrm{ki}}} + \frac{\partial \mathcal{W}_{\mathrm{ki}}}{\partial \theta_{\mathrm{ki}}} \\
&= -\frac{1}{\sigma^2} + \sum_{n=1}^{N} w_{\mathrm{ki}} \left(x_i^n - \alpha e^{w_{\mathrm{ki}}}\right) \big(\Theta\{h_k^n\} - S_k\left(t\right)\big) \\
&+ \sum_{n=1}^{N} w_{\mathrm{ki}}^2 \Big\{-\alpha e^{w_{\mathrm{ki}}} \big(\Theta\{h_k^n\} - S_k\left(t\right)\big) - \left(x_i^n - \alpha e^{w_{\mathrm{ki}}}\right)^2 S_k\left(t\right) \left(1 - S_k\left(t\right)\right)\Big\} \\
&+ \sum_{n=1}^{N} \big(w_{\mathrm{ki}} \alpha e^{w_{\mathrm{ki}}} + w_{\mathrm{ki}}^2 \alpha e^{w_{\mathrm{ki}}}\big) \left(S_k\left(t\right) - \Theta\{h_k^n\}\right) \\
&= -\frac{1}{\sigma^2} + \sum_{n=1}^{N} w_{\mathrm{ki}} \left(x_i^n - \alpha e^{w_{\mathrm{ki}}}\right) \big(\Theta\{h_k^n\} - S_k\left(t\right)\big) + \sum_{n=1}^{N} w_{\mathrm{ki}} \alpha e^{w_{\mathrm{ki}}} \left(S_k\left(t\right) - \Theta\{h_k^n\}\right)
\end{aligned} \tag{S8}$$

According to the zero gradient, we can further get,

$$\sum_k \sum_i \nabla^2 \widetilde{f}\left(\theta_{\mathrm{ki}}\right) = -\frac{\mathrm{KI}}{\sigma^2} + \sum_k \sum_i \frac{1}{\sigma^2} \left(\theta_{\mathrm{ki}} - \mu\right) + \sum_k \sum_i \sum_n w_{\mathrm{ki}} \alpha e^{w_{\mathrm{ki}}} \left(S_k\left(t\right) - \Theta\{h_k^n\}\right) \tag{S9}$$

It is obvious that the trace of Hessian matrix consists of three terms: $-\frac{KI}{\sigma^2}$, $\sum_k \sum_i \frac{1}{\sigma^2}(\theta_{ki} - \mu)$, $\sum_n \sum_k \sum_i w_{ki} \alpha e^{w_{ki}} (S_k(t) - \Theta\{h_k^n\})$. Whether the trace is positive will be analyzed through two steps.

First, we will illustrate that the second term is much smaller than the third. When the network is stable, i.e., the gradient is zero, by considering a large number of samples, we will get:

$$\sum_k \sum_i \frac{(\theta_{ki} - \mu)}{N\sigma^2 w_{ki} \alpha e^{w_{ki}} (S_k(t) - \Theta\{h_k^n\})} = \sum_k \sum_i (\frac{\sum_n x_i^n}{N} - \alpha e^{w_{ki}}) \tag{S10}$$

Under the theoretically optimal STDP learning rule, the following equation will be derived (Habenschuss et al., 2013; Nessler et al., 2009):

$$\alpha e^{w_{ki}} < x_i >_{\tilde{p}(\boldsymbol{x}|z_k=1,w)} = 1 \tag{S11}$$

This means that $\frac{\sum_n x_i^n}{N} - \alpha e^{w_{ki}} \approx 0$. Through the above two points, we can derive that

$$\begin{aligned} \sum_k \sum_i \frac{(\theta_{ki} - \mu)}{N\sigma^2 w_{ki} \alpha e^{w_{ki}} (S_k(t) - \Theta\{h_k^n\})} &\approx \frac{\sum_k \sum_i (\theta_{ki} - \mu)}{\sum_k \sum_i N\sigma^2 w_{ki} \alpha e^{w_{ki}} (S_k(t) - \Theta\{h_k^n\})} \\ &\approx 0 \end{aligned} \tag{S12}$$

That is to say, the second term is much smaller than the third: $\frac{1}{\sigma^2} \sum_k \sum_i (\theta_{ki} - \mu) \ll N \sum_k \sum_i w_{ki} \alpha e^{w_{ki}} (S_k(t) - \Theta\{h_k^n\})$.

Second, we will discuss whether the third term is positive. For each sample in the Winner-Take-All networks, the third term can be split into two parts: one part represents neuron z_k which actually release spikes, one part represents expected neuron z_{lable}:

$$N(\sum_i w_{ki} \alpha e^{w_{ki}} - \sum_i w_{lable,i} \alpha e^{w_{lable,i}}) \tag{S13}$$

According to the theoretically optimal STDP learning rule Eq.S11, the expression will become the approximate difference of actual and expected neurons: $N(\sum_i w_{ki} x_i - \sum_i w_{lable,i} x_i)$. When the network is trapped in the saddle points, the neuron which releases spikes is not the expected. We can get that the potential of the actual neuron is higher than the expected:$u_k > u_{lable} \Rightarrow \sum_i w_{ki} x_i > \sum_i w_{lable,i} x_i$. As a result, the third term is always positive when the network is trapped in the saddle points.

In summary, as long as the third term is large enough, the first and second term can be ignored, and the trace of Hessian matrix will be positive so that strict saddle property will be satisfied.

2 PROOF OF THEOREM 2

Under the condition that $x|\theta \sim \mathcal{N}(\mu, \sigma^2)$ and noise $\varepsilon \sim \mathcal{N}(0,1)$, the aim is to get $p(\varepsilon|\theta)$. By inducing hidden variable x, we get

$$p(\varepsilon|\theta) = \int p(\varepsilon|x)\, p(x|\theta) \mathrm{dx} \tag{S14}$$

Given the input x, the distribution of noise ε can be regarded as that of a mixture of noise and input. We get

$$p\left(\varepsilon|\theta\right) \propto \int p(\varepsilon + x)\ p\left(x|\theta\right)\mathrm{dx} \tag{S15}$$

According to Taylor expansion $p\left(x+\varepsilon\right) = p(x) + \varepsilon p^{'}(x) + \frac{1}{2}\varepsilon^2 p^{''}(x) + o(\varepsilon^3)$, where $p^{'}\left(x|\theta\right) = -\frac{x}{\sigma^2}p\left(x|\theta\right)$, $p^{''}\left(x|\theta\right) = -p\left(x|\theta\right)\left(\frac{1}{\sigma^2} - \frac{x^2}{\sigma^4}\right)$, we get

$$\begin{aligned} p\left(\varepsilon|\theta\right) &\propto \textstyle\int \left(p(x|\theta) + \varepsilon p^{'}(x|\theta) + \frac{1}{2}\varepsilon^2 p^{''}(x|\theta)\right)\ p\left(x|\theta\right)\mathrm{dx} \\ &\propto \textstyle\int \left(1 - \frac{\varepsilon x}{\sigma^2} - \frac{\varepsilon^2}{2}\left(\frac{1}{\sigma^2} - \frac{x^2}{\sigma^4}\right)\right)\ p\left(x|\theta\right)\mathrm{dx} \end{aligned} \tag{S16}$$

As Taylor approximation $e^{-\frac{\varepsilon x}{\sigma^2} - \frac{\varepsilon^2}{2}\left(\frac{1}{\sigma^2} - \frac{x^2}{\sigma^4}\right)} = 1 - \frac{\varepsilon x}{\sigma^2} - \frac{\varepsilon^2}{2}\left(\frac{1}{\sigma^2} - \frac{x^2}{\sigma^4}\right)$, we get

$$p\left(\varepsilon|\theta\right) \propto \int e^{-\frac{\varepsilon^2}{2\sigma^2} - \frac{x^2}{\sigma^2} + \frac{\varepsilon x}{\sigma^2} + \frac{\varepsilon^2}{2}\left(\frac{1}{\sigma^2} - \frac{x^2}{2\sigma^4}\right)}\mathrm{dx} \tag{S17}$$

The exponential term can be arranged as

$$-\frac{\varepsilon^2}{2\sigma^2} - \frac{x^2 + \frac{2\sigma^2\varepsilon x}{\sigma^2-\varepsilon^2} + \left(\frac{\sigma^2\varepsilon}{\sigma^2-\varepsilon^2}\right)^2}{\frac{2\sigma^4}{\sigma^2-\varepsilon^2}} + \frac{\varepsilon^2}{2(\sigma^2-\varepsilon^2)} \tag{S18}$$

The second term is binomial of x and in fact is some constant with the integration of x. The numerator and denominator of the last term is the same order of ε and can also regard as some constant. We also verify the approximation between $e^{-\frac{\varepsilon^2}{2\sigma^2}}ande^{-\frac{\varepsilon^2}{2\sigma^2} + \frac{\varepsilon^2}{2(\sigma^2-\varepsilon^2)}}$ in Matlab simulations. Results also show that these two functions are nearly the same. We finally improve that

$$p\left(\varepsilon|\theta\right) \propto e^{-\frac{\varepsilon^2}{2\sigma^2}} \tag{S19}$$

The previous work (Habenschuss et al., 2013; Kappel et al., 2015) shows that in the spiked-based WTA networks, one prominent motif of cortical microcircuits, $p\left(x|\theta\right)$ is the integration of N Poisson distribution with the mean $\alpha e^{w_{\mathrm{ki}}}$, which can approximate Normal distribution $\mathcal{N}\left(N\alpha e^{w_{\mathrm{ki}}}, N\alpha e^{w_{\mathrm{ki}}}\right)$. Therefore, we get,

$$\varepsilon|\theta \sim \mathcal{N}\left(0, N\alpha e^{w_{\mathrm{ki}}}\right) \tag{S20}$$

3 BACK-PROPAGATION FOR SYNAPTIC SAMPLING ON THE THREE-LAYER NETWORK.

In this section we derive learning rules based on back-propagation for synaptic sampling. As shown in Fig.S1, we add one hidden layer $\boldsymbol{z^n}$ to the two-layer network. In the two-layer model, given $y^n = k$, input $\boldsymbol{x_i^n}$ follows a Poisson distribution with a mean that is affected by synaptic weight

w_{ki}. The firing rate is proportional to the posterior probability $p(z^n = k|x^n, w)$. In the three-layer network, such a relationship becomes the product of Poisson distribution. Assuming that $A_{\text{kj}} = POISSON(y_j^n|\alpha e^{w_{\text{kj}}})$ $B_{\text{ji}} = POISSON(x_i^n|\alpha e^{w_{\text{ji}}})$, one obtains the posterior probability of corresponding spiking neuron k as

$$p(z^n = k|x^n, w) = \frac{\prod_i \sum_j A_{\text{kj}} B_{\text{ji}}}{\sum_k \prod_i \sum_j A_{\text{kj}} B_{\text{ji}}} \tag{S21}$$

The likelihood function becomes

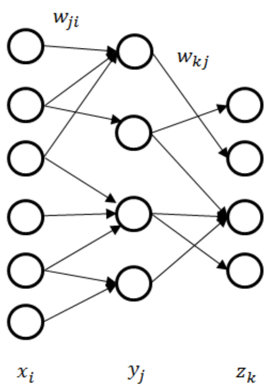

Figure S1. A three-layer neural network diagram.

$$\begin{aligned}\log p_N(J|\theta) &= \sum_{n=1}^{N}\sum_{k=1}^{K} \Theta\{h_k^n\} \log p(z^n = k|x^n, w) \\ &= \sum_{n=1}^{N}\sum_{k=1}^{K} \Theta\{h_k^n\} \log \frac{\prod_i \sum_j \text{POISSON}\left(x_i^n|\alpha e^{w_{\text{ji}}}\right) POISSON(y_j^n|\alpha e^{w_{\text{kj}}})}{\sum_k \prod_i \sum_j \text{POISSON}\left(x_i^n|\alpha e^{w_{\text{ji}}}\right) POISSON(y_j^n|\alpha e^{w_{\text{kj}}})}\end{aligned} \tag{S22}$$

The derivation of synaptic weight w_{kj} of the second layer is

$$\frac{\partial \log p_N(J|\theta)}{\partial w_{\text{kj}}} = \sum_{n=1}^{N} \frac{\partial(log \prod_i \sum_j A_{\text{kj}} B_{\text{ji}} - log \sum_k \prod_i \sum_j A_{\text{kj}} B_{\text{ji}})}{\partial w_{\text{kj}}} \tag{S23}$$

In Eq.S21, the deviation of numerator and denominator is

$$\begin{aligned}\frac{\partial \prod_i \sum_j A_{\text{kj}} B_{\text{ji}}}{\partial w_{\text{kj}}} &= B_{j1} A_{\text{kj}} \prod_{i\neq 1}\sum_j A_{\text{kj}} B_{\text{ji}} \left(y_j^n - \alpha e^{w_{\text{kj}}}\right) \\ &+ B_{j2} A_{\text{kj}} \prod_{i\neq 2}\sum_j A_{\text{kj}} B_{\text{ji}} \left(y_j^n - \alpha e^{w_{\text{kj}}}\right) + \ldots \\ &\approx M \prod_i \sum_j A_{\text{kj}} B_{\text{ji}} \left(y_j^n - \alpha e^{w_{\text{kj}}}\right)\end{aligned} \tag{S24}$$

$$\frac{\partial \sum_k \prod_i \sum_{z_j^n} A_{\mathrm{kj}} B_{\mathrm{ji}}}{\partial w_{\mathrm{kj}}} = \frac{\partial \prod_i \sum_j A_{\mathrm{kj}} B_{\mathrm{ji}}}{\partial w_{\mathrm{kj}}} \tag{S25}$$

When M is large enough, derive the log of numerator and denominator and we obtain

$$\frac{\partial \log \prod_i \sum_j A_{\mathrm{kj}} B_{\mathrm{ji}}}{\partial w_{\mathrm{kj}}} \approx M \left(y_j^n - \alpha e^{w_{\mathrm{kj}}} \right) \tag{S26}$$

$$\frac{\partial \log \sum_k \prod_i \sum_j A_{\mathrm{kj}} B_{\mathrm{ji}}}{\partial w_{\mathrm{kj}}} \approx Mp\left(z^n = k | x^n, w\right) \left(y_j^n - \alpha e^{w_{\mathrm{kj}}} \right) \tag{S27}$$

Learning rule for the second layer is derived as

$$d\theta_{kj} \quad = b\left(\tfrac{1}{\sigma^2}\left(\mu - \theta_{\mathrm{ki}}\right) + \textstyle\sum_{n=1}^{N} (w_{kj} \left(y_j^n - \alpha e^{w_{\mathrm{kj}}} \right) \left(\Theta\{h_k^n\} - S_k\left(t\right)\right)\right) dt + bd\mathcal{W}_{\mathrm{kj}} \tag{S28}$$

The derivation of synaptic weight w_{ji} of the first layer is

$$\frac{\partial \log p_N\left(J|\theta\right)}{\partial w_{\mathrm{ji}}} = \sum_{n=1}^{N} \frac{\partial \log p_N\left(J|\theta\right)}{\partial y_j^n} \frac{\partial y_j^n}{\partial w_{\mathrm{ji}}} \tag{S29}$$

First, we derive the second part $\frac{\partial z_j^n}{\partial w_{\mathrm{ji}}}$. In the spike-based neural networks, the firing rate of stochastic spiking neurons depends exponentially on the membrane voltage. It has been proposed that the exponential relationship between the membrane potential and the firing rate is a good approximation to the firing characteristics of cortical pyramidal neurons (Jolivet et al., 2006).The membrane voltage $u_j(t)$ of neuron j in the two-layer network is given by

$$u_j^n = \sum_i w_{\mathrm{ji}}^1 x_i^n + b_j^1 \tag{S30}$$

The corresponding instantaneous firing rate $\rho_j(t)$ of neuron j is given by

$$\rho_j\left(t\right) = \frac{\rho_{\mathrm{net}}}{I_{\mathrm{lat}}} e^{u_j(t)} \tag{S31}$$

where $I_{\mathrm{lat}}(t)$ is divisive lateral inhibition, i.e. $I_{\mathrm{lat}}\left(t\right) = \sum_l e^{u_l(t)}$. Then we get,

$$\begin{aligned} \frac{\partial y_j^n}{\partial w_{\mathrm{ji}}} = \frac{\partial \rho_j(t)}{\partial w_{\mathrm{ji}}} &= \rho_{\mathrm{net}} \left(\frac{\sum_{l \neq k} e^{u_l(t)}}{\left(I_{\mathrm{lat}}\right)^2} \right) \frac{\partial e^{u_j(t)}}{\partial w_{\mathrm{ji}}} \\ &\propto \frac{\rho_{\mathrm{net}}}{I_{\mathrm{lat}}} e^{u_j(t)} \frac{\partial u_j\left(t\right)}{\partial w_{\mathrm{ji}}} = \rho_j(t) x_i^n \\ &\propto x_i^n \end{aligned} \tag{S32}$$

In the WTA neural circuit, when neuron j spikes, $y_j^n(t) = 1$. Thus we get the approximation of last term. Then we derive the first part $\frac{\partial \log p_N(J|\theta)}{\partial y_j^n}$. Under the theoretically optimal STDP learning rule, the following equation will be derived(Habenschuss et al., 2013; Nessler et al., 2009):

$$E\left(y_j^n\right) = \alpha e^{w_{\mathrm{kj}}} \tag{S33}$$

We can get

$$\begin{aligned} \frac{\partial \log p_N\left(J|\theta\right)}{\partial y_j^n} &= \sum_{m,q} \frac{\partial \log p_N\left(J|\theta\right)}{\partial w_{\mathrm{mq}}} \frac{\partial w_{\mathrm{mq}}}{\partial y_j^n} \\ &= \sum_{m} \frac{\partial \log p_N\left(J|\theta\right)}{\partial w_{mj}} \frac{\partial w_{\mathrm{mj}}}{\partial y_j^n} \\ &= \frac{\alpha}{E(y_j^n)} \left(\frac{d\theta_{\mathrm{kj}}}{w_{\mathrm{kj}}} + \frac{d\theta_{\mathrm{tj}}}{w_{\mathrm{tj}}} \right) \end{aligned} \tag{S34}$$

The last equation in Eq.S34 holds because when spiking networks finish one weight update, only the actual neuron i and expected neuron k update corresponding weights in the WTA circuits. Substitute Eq.S32 and Eq.S34 to Eq.S29. Assuming that $\beta = \frac{1}{E\left(z_j^n\right)}$, apply the chain rule and we get,

$$\begin{aligned} d\theta_{\mathrm{ji}} &= \frac{\partial logp_N\left(J|\theta\right)}{\partial w_{\mathrm{ji}}} \frac{\partial w_{\mathrm{ji}}}{\partial \theta_{\mathrm{ji}}} \\ &= (\sum_{n=1}^{N} \frac{\partial logp_N\left(J|\theta\right)}{\partial y_j^n} \frac{\partial y_j^n}{\partial w_{\mathrm{ji}}}) \frac{\partial w_{\mathrm{ji}}}{\partial \theta_{\mathrm{ji}}} \\ &= \left(\beta\alpha \sum_{n=1}^{N} x_i^n \left(\frac{d\theta_{\mathrm{kj}}}{w_{\mathrm{kj}}} + \frac{d\theta_{\mathrm{tj}}}{w_{\mathrm{tj}}} \right) \right) * w_{\mathrm{ji}} \end{aligned} \tag{S35}$$

Note that the synaptic weights of two layers is about the same order of magnitude and hence cancel each other. We get the compact approximation for learning rule in the first layer,

$$d\theta_{\mathrm{ji}} \approx \beta\alpha \sum_{n=1}^{N} x_i^n \left(d\theta_{\mathrm{kj}} + d\theta_{\mathrm{tj}} \right) \tag{S36}$$

In fact, the experiment results also show the performance of compact learning rules in Eq.S36 is as good as the exact one in Eq.S35.

Similarly, the learning rule for L-layer spiking neural networks can be concluded as follow. Given the L-layer noisy spiking neural networks, each layer computes a function $X^l = g_l(X^{l-1}, W^l)$, where X^l is the output of the l_{th} layer, $X^{l-1}$is the input of the l_{th} layer and W^l is the vector of adjustable parameters between the $(l-1)_{th}$ and the l_{th} layer. Note the vector X^1 in the first layer is the input sample. The learning rule for L-layer spiking neural networks can be concluded as follow.

$$d\theta_{kj}^{L} \quad = b\left(\frac{1}{\sigma^2}\left(\mu - \theta_{\mathrm{kj}}^{L}\right) + \textstyle\sum_{n=1}^{N}(w_{kj}^{L}\left(x_{j}^{n,L-1} - \alpha e^{w_{\mathrm{kj}}^{L}}\right)\left(\Theta\{h_{k}^{n}\} - S_{k}^{L}\left(t\right)\right)\right)\right) dt + bd\mathcal{W}_{\mathrm{kj}}^{L} \quad \text{(S37)}$$

$$d\theta_{\mathrm{ji}}^{l-1} = \beta\alpha \sum_{n=1}^{N} x_{i}^{n,l-1}\left(d\theta_{\mathrm{kj}}^{l} + d\theta_{\mathrm{mj}}^{l}\right)\ (2 \leq l \leq L) \quad \text{(S38)}$$

where $d\theta_{\mathrm{kj}}^{l}$ represents change in parameters corresponding to the neuron k which releases a spike and $d\theta_{\mathrm{mj}}^{l}$ represents change in parameters corresponding to the desired neuron m.

REFERENCES

Habenschuss, S., Puhr, H., and Maass, W. (2013). Emergence of optimal decoding of population codes through STDP. Neural Computation 25, 1371–1407

Jolivet, R., Rauch, A., Lüscher, H.-R., and Gerstner, W. (2006). Synaptic modifications in cultured hippocampal neurons: Dependence on spike timing, synaptic strength, and postsynaptic cell type. Journal of Computational Neuroscience 21, 35–49

Kappel, D., Habenschuss, S., Legenstein, R., and Maass, W. (2015). Network plasticity as Bayesian inference. PLoS Computational Biology 11, e1004485

Nessler, B., Pfeiffer, M., and Maass, W. (2009). STDP enables spiking neurons to detect hidden causes of their inputs. In Advances in Neural Information Processing Systems 22 (Vancouver, British Columbia, Canada). 1357–1365
